# Gut microbiota and fecal metabolic signatures in rat models of disuse-induced osteoporosis

**DOI:** 10.3389/fcimb.2022.1018897

**Published:** 2022-12-14

**Authors:** Xiaochen Qiao, Kun Zhang, Xiaoyan Li, Zhi Lv, Wenhao Wei, Ruhao Zhou, Lei Yan, Yongchun Pan, Sen Yang, Xiaojuan Sun, Pengcui Li, Chaojian Xu, Yi Feng, Zhi Tian

**Affiliations:** ^1^ Second Clinical Medical College, Shanxi Medical University, Taiyuan, Shanxi, China; ^2^ Department of Orthopedics, The Second Hospital of Shanxi Medical University, Shanxi Key laboratory of Bone and Soft Tissue Injury Repair, Taiyuan, Shanxi, China; ^3^ Department of Orthopedics, JinZhong Hospital Affiliated to Shanxi Medical University, Jinzhong, Shanxi, China; ^4^ Shanxi Province Cancer Hospital, Shanxi Hospital Affiliated to Cancer Hospital, Chinese Academy of Medical Sciences, Cancer Hospital Affiliated to Shanxi Medical University, Taiyuan, Shanxi, China; ^5^ Department of Orthopedics, Third People’s Hospital of Datong City, Datong, Shanxi, China; ^6^ Department of Orthopedics, The Second People’s Hospital of Changzhi, Changzhi, Shanxi, China

**Keywords:** osteoporosis, disuse, gut microbiota, 16s rDNA sequencing, metabolomics

## Abstract

**Background:**

Assessing the correlation between gut microbiota (GM) and bone homeostasis has increasingly attracted research interest. Meanwhile, GM dysbiosis has been found to be associated with abnormal bone metabolism. However, the function of GM in disuse-induced osteoporosis (DIO) remains poorly understood. In our research, we evaluated the characteristics of GM and fecal metabolomics to explore their potential correlations with DIO pathogenesis.

**Methods:**

DIO rat models and controls (CON) underwent micro-CT, histological analyses, and three-point bending tests; subsequently, bone microstructures and strength were observed. ELISAs were applied for the measurement of the biochemical markers of bone turnover while GM abundance was observed using 16S rDNA sequencing. Metabolomic analyses were used to analyze alterations fecal metabolites. The potential correlations between GM, metabolites, and bone loss were then assessed.

**Results:**

In the DIO group, the abundance of GM was significantly altered compared to that in the CON group. Moreover, DIO significantly altered fecal metabolites. More specifically, an abnormally active pathway associated with bile acid metabolism, as well as differential bacterial genera related to bone/tissue volume (BV/TV), were identified. Lithocholic acid, which is the main secondary bile acid produced by intestinal bacteria, was then found to have a relationship with multiple differential bacterial genera. Alterations in the intestinal flora and metabolites in feces, therefore, may be responsible for DIO-induced bone loss.

**Conclusions:**

The results indicated that changes in the abundance of GM abundance and fecal metabolites were correlated with DIO-induced bone loss, which might provide new insights into the DIO pathogenesis. The detailed regulatory role of GM and metabolites in DIO-induced bone loss needs to be explored further.

## Introduction

Osteoporosis is the most common bone disorder and is defined as a systemic skeletal disease characterized by low bone mass and the microarchitectural deterioration of bone tissue. It further leads to increased bone fragility and susceptibility to fractures (Locantore et al., 2020; Kim et al., 2021). The incidence of osteoporotic fractures is rapidly increasing in the aging population. Indeed, osteoporotic fractures seriously impact the quality of life and mortality of patients, as well as overall healthcare costs (Pisani et al., 2016).

Osteoporosis can be divided into primary and secondary osteoporosis depending on its etiology. Disuse-induced osteoporosis (DIO), which is a type of secondary osteoporosis with presently unsatisfactory treatment options, is a common complication caused by the lack, or disuse, of mechanical loading or systemic immobilization and constitutes a state of bone loss (Yang et al., 2020; Rolvien and Amling, 2022). Currently, drug therapy remains the primary treatment for osteoporosis (Ou et al., 2021). However, the long-term use of these drugs can lead to serious complications, including kidney damage, venous thrombosis, and an increased risk of developing tumors (Ou et al., 2021). Thus, more effective, safe, and novel treatment strategies are urgently required for DIO.

Gut microbiota (GM), referred to as the “second gene pool,” comprise a collection of microorganisms that colonize the gastrointestinal tract (Qin et al., 2010; Tu et al., 2021). GM homeostasis is implicated in many aspects of human health, including neurological disorders, abnormal inflammatory responses, and metabolic diseases (Cani, 2018; Needham et al., 2020; Suganya and Koo, 2020; Yoo et al., 2020). Osteoporosis, which is a systemic metabolic bone disease, is closely related to the GM. In a study by Wen, a postmenopausal osteoporosis mouse model was constructed and revealed that *Ruminococcus flavefaciens* exhibited the greatest variation in abundance among the GM and was also associated with osteoclastic indicators and the estrobolome (Wen et al., 2020). Ma further carried out a metabolomics analysis of serum and fecal metabolites in animal models of postmenopausal osteoporosis and found that changes in the GM, as well as fecal and serum metabolites were responsible for the occurrence and development of postmenopausal osteoporosis (Ma et al., 2020b). Ma also observed an association between the composition and function of GM and senile osteoporosis in an aged rat model, demonstrating that variations in GM may contribute to senile osteoporosis through metabolic pathways (Ma et al., 2020a). In addition, glucocorticoid-, alcohol-, and high-fat-induced osteoporosis are all associated with dysbacteriosis in the GM (Schepper et al., 2020; Cheng et al., 2021; Lu et al., 2021).

To date, the GM, metabolites, and mechanisms by which the GM affect DIO remain poorly understood. In the present study, we constructed a DIO rat model and applied an integrated approach comprising 16S rDNA gene sequencing combined with fecal ultra-high-performance liquid chromatography-mass spectrometry to elucidate the association between GM and DIO. Understanding the characteristics of GM and GM-derived metabolites in DIO may provide insights that will contribute to the development, prevention, and treatment of osteoporosis.

## Materials and methods

### Animals

Twelve-week-old female Sprague-Dawley rats were purchased from the Ying Ze District Campus Animal Testing Center at Shanxi Medical University. The rats were housed in a non-specific microbial environment with a constant temperature of 23 ± 2°C, 12-h light/dark cycle, and allowed ad libitum access to sterile food and autoclaved water. After one week acclimatization, the rats were randomly divided into DIO and control (CON) groups (n = 6/group). Right leg sciatic neurotomies were performed on the rats to construct DIO models; the same amount of adipose tissue was taken from both groups (Monzem et al., 2021). The animals were sacrificed 10 weeks post-operation, and target samples were collected from each group. All animal experiments were performed in strict accordance with the National Institutes of Health (NIH) Guidelines for the Care and Use of Experimental Animals and were approved by the Ethical Committee of Experimental Animal Care of Shanxi Medical University (permit number: 2021014).

### Micro-CT scanning

Distal femurs were scanned at a high resolution using Micro-CT (vivaCT80, Scanco, Switzerland) to analyze differences in the volumes and structures of the trabecular bones between the two groups. We manually selected 100 contiguous cross-sectional slices above the limit of the femoral growth plate to analyze the bones. Histomorphometric parameters were computed using Scanco Medical software. The bone mineral density (BMD), bone volume per tissue volume (BV/TV), trabecular number (Tb.N), trabecular thickness (Tb.Th), cortical bone thickness (Ct.Th), and cortical bone volume (Ct.V), were determined for each sample.

### Histological analysis

The femoral samples were fixed with 4% paraformaldehyde for 24 h and decalcified in 20% ethylenediaminetetraacetic acid solution at 37°C for 6–7 weeks until the femurs had softened. The femurs were then dehydrated, embedded in paraffin, cut into 5-mm longitudinal sections, dried, and stained with hematoxylin and eosin. Their morphological characteristics were examined using light microscopy.

### Three-point bending test

The femurs were subjected to a three-point bending test. The mechanical properties of the femurs were evaluated at the mid-diaphysis using an electronic universal testing machine (ElectroForce 3200 Series, TA Instruments, USA). The femoral samples were centered and fixed on a three-point bending test stent with two fixed loading points separated by a 20-mm gap. A bending load was applied at a constant displacement rate of 3 mm/min until fractures occurred. The internal and external major and minor axis lengths of the femurs at the fracture points were then measured. The following parameters of the samples were obtained: peak load, fracture load, maximum displacement, and stiffness.

### ELISA

Blood samples were collected from the hearts of rats and centrifuged for 15 min at 3,000 rpm to separate the sera. The abundance of the N-terminal propeptide of type I procollagen (PINP) and C-terminal telopeptide of type I collagen (CTX-I) was measured using ELISA kits (Lunchang Shuo Biotechnology, Xiamen, China) according to the manufacturer’s protocol.

### Feces collection

Fecal samples were directly collected from the rats in both groups. At least two fecal pellets were collected from each rat: one was used for microbial analysis, while the other was subjected to metabolic analysis. The fecal samples were placed in sterile centrifuge tubes, frozen in liquid nitrogen immediately, and stored at −80°C for further sequencing.

### 16S rDNA sequencing

16S rDNA sequencing was carried out at Lc-Bio Technologies Co., Ltd. DNA from different samples were extracted using the cetyltrimethylammonium ammonium bromide (CTAB) method. We selected the V3–V4 region of the 16S rRNA gene using the custom barcode universal bacterial primers 341F (5’-CCTACGGGNGGCWGCAG-3’) and 805R (5’-GACTACHVGGGTATCTAATCC-3’). The PCR products were confirmed with 2% agarose gel electrophoresis and purified using AMPure XT beads (Beckman Coulter Genomics, Danvers, USA), and quantified by Qubit (Invitrogen, California, USA). The purified PCR products were assessed on an Agilent 2100 Bioanalyzer (Agilent, California, USA) and the Library Quantification Kit for Illumina (Kapa Biosciences, Woburn, USA). The libraries were sequenced on the NovaSeq 6000 platform (Illumina, San Diego, CA, USA).

### 16S rDNA microbial community analysis

Paired-end reads were assigned to samples based on their unique barcodes and truncated by cutting off the barcode and primer sequence, and merged using FLASH (v1.2.8, http://ccb.jhu.edu/software/FLASH/). Quality filtering of the raw reads was performed to obtain high-quality clean tags using fqtrim (v0.94, http://ccb.jhu.edu/software/fqtrim/). Chimeric sequences were filtered using Vsearch (v2.3.4, https://github.com/torognes/vsearch). Subsequently, we performed DADA2 analysis using QIIME2 (v2019.7, https://qiime2.org/) to obtain Amplicon Sequence Variant (ASV) tables and sequences. Alpha diversity and beta diversity were analyzed based on the above ASV tables and sequences. Lastal+ (v2017.3, https://github.com/hallamlab/LAST-Plus/wiki) was used for sequence alignment, and the feature sequences were annotated with the NT-16s database for each representative sequence. Finally we conducted difference analysis and advanced analysis between two groups. The graphs were drawn using R (v3.5.2).

### Extraction and UHPLC-MS/MS analysis of fecal metabolites

The collected samples were thawed on ice, and metabolites were extracted with 50% methanol buffer. Metabolite-containing supernatants were obtained after centrifugation at 4,000 g for 20 min and stored at -80°C prior to LC-MS analysis. In addition, pooled QC samples were prepared by combining 10 μL of each extraction mixture. We ensured that different groups were cross-sorted on the machine. All chromatographic separations were performed using a Thermo Scientific UltiMate 3000 HPLC system (Thermo Scientific, Waltham, USA). A high-resolution tandem mass spectrometer Q-Exactive (Thermo Scientific, Waltham, USA) was used to collect first and secondary order spectrum data of the metabolites eluted from the column, and operated in both the positive and negative ion modes. To evaluate the stability of the LC-MS during the data acquisition stage, a quality control sample was acquired after every 10 samples.

### Bioinformatics of fecal metabolome data

Pretreatments of the acquired mass spectrum raw data were performed using Compound Discoverer 3.1.0 (Thermo Fisher Scientific, Waltham, USA). Each ion was identified by combining retention time (RT) and m/z data. We use the chemspider database as a plug-in for database search using the Compound Discoverer software. Using online Kyoto Encyclopedia of Genes and Genomes (KEGG) and Human Metabolome Database (HMDB), the metabolites were annotated by matching their molecular weights (mass difference less than 10 ppm), substance names and formulas. The intensity of peak data was further preprocessed using metaX (v1.4.16, https://www.ncbi.nlm.nih.gov/pubmed/28327092). The features that were detected in less than 50% of Quality Control (QC) samples or 80% of biological samples were removed, and the remaining peaks with missing values were imputed with the k-nearest neighbor algorithm to further improve data quality. Principal Component Analysis (PCA) was performed for outlier detection using the pre-processed dataset. Probabilistic Quotient Normalization (PQN) was used to normalize the data to obtain the normalized ion intensity data of each sample. In addition, the Coefficient of Variations of the metabolic features were calculated across all QC samples, and those > 30% were then removed. The P-values were adjusted for multiple tests using the False Discovery Rate and Benjamini-Hochberg method. Supervised Partial Least Squares-Discriminant Analysis (PLS-DA) was conducted using metaX (v1.4.16, https://www.ncbi.nlm.nih.gov/pubmed/28327092) to discriminate the different variables between two groups, and Variable Influence of Projection (VIP) values were calculated. A VIP cut-off value of 1.0 was used to select important features. KEGG enrichment analysis was performed on significantly different metabolites. The correlations between differential bacterial genera, bone phenotypes, and metabolites were analyzed using R (v3.5.2).

### Statistical analysis

The statistical significance of bone mass and biochemical indices was assessed using Student’s *t*-tests or a one-way analysis of variance in GraphPad Prism (v.9.0). Spearman’s rank correlation was applied for the association analysis of the 16S microbiome, bone phenotypes and fecal metabolites. Differences were considered statistically significant at P < 0.05.

## Results

### DIO-induced bone loss in rats

**Figure 1:**
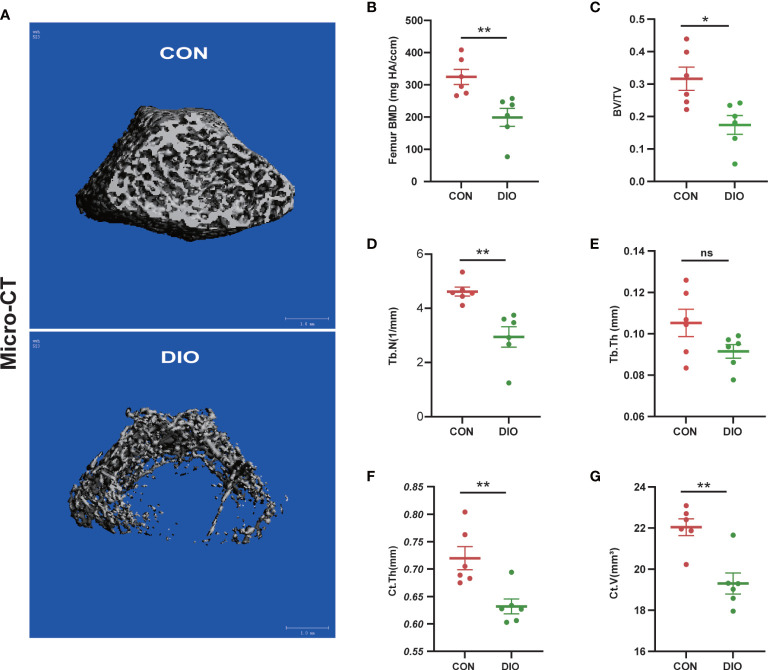
DIO-induced bone loss in rats. **(A)** Representative Micro-CT 3D reconstructions of the two groups. **(B–E)** Trabecular bone parameters at the distal femoral metaphysis after 10 weeks, including BMD, BV/TV, Tb.N, and Tb.Th. **(F, G)** Cortex bone parameters at the middle femur after 10 weeks, including Ct.Th and Ct.V. Data are expressed as the mean ± SEM. n = 6, *P < 0.05, **P < 0.01, ns, no significance.

Micro-CT was used to analyze distal femur trabecular bone microstructures. The associated 3D structures (BMD, BV/TV, Tb.N, Tb.Th, Ct.Th, and Ct.V) revealed that the distal femur trabecular bone microstructure was significantly destroyed in the DIO group compared with that in the CON group (**Figure 1A**). Moreover, distinct reductions in BMD, BV/TV, Tb.N, Ct.Th, and Ct.V were observed. (**Figures 1B–D, F, G**). However, there were no significant differences in the Tb.Th between the two groups (**Figure 1E**). These results indicate that DIO causes marked femoral bone loss.

### Changes in bone morphology, reductions in bone mechanical properties, and inhibition of bone formation in DIO

**Figure 2:**
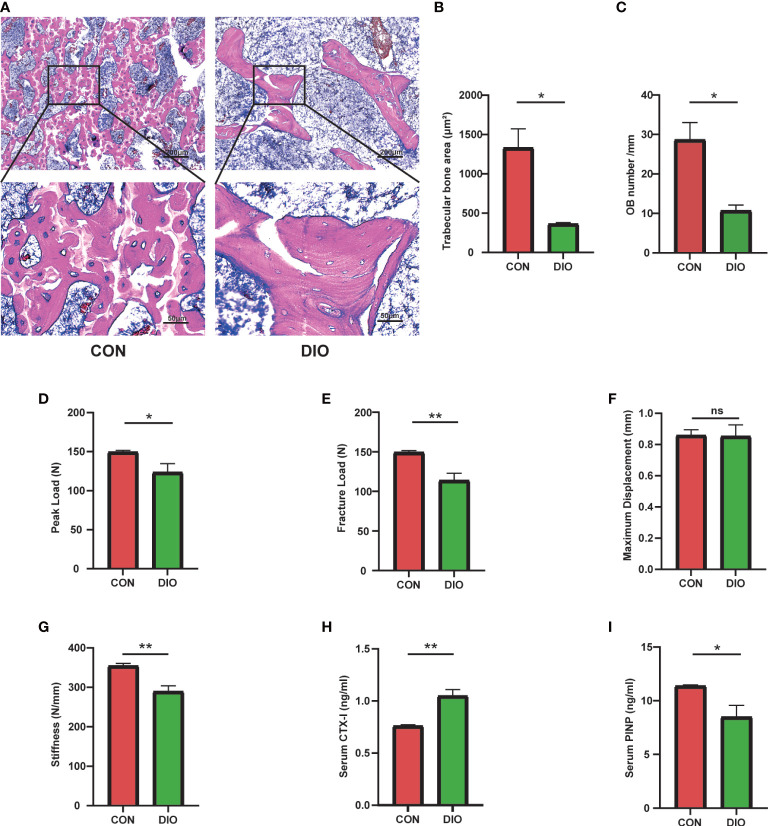
Changes in bone morphology, reduced bone mechanical properties, and inhibition of bone formation in DIO. **(A)** The bone tissue of distal femoral metaphysis was observed by H&E staining. **(B)** Trabecular bone area ratio. **(C)** Osteoblast numbers. **(D–G)** Parameters of the three-point bending test, including the peak load, fracture load, maximum displacement, and stiffness. **(H, I)** Serum levels of bone turnover biomarkers, including CTX-I and PINP. Data are expressed as the mean ± SEM. n = 6, *P < 0.05, **P < 0.01, ns, no significance.

To further determine the changes in bone morphology in rats with DIO, histopathological examinations were performed on bone tissues. Compared with that in the CON group, the trabecular bone in the DIO group became sparse, and its shape changed from plate- to rod-like (**Figure 2A**). We further observed a marked decrease in the area ratios of the trabecular bone (**Figure 2B**) and osteoblasts (**Figure 2C**) in the DIO-induced rats. Additionally, we tested the mechanical properties of the femurs using a three-point bending test. The peak load, fracture load, and stiffness of the disused femurs were significantly decreased compared to those in the CON group, and no significant difference was observed in the maximum displacement between the two groups (**Figures 2D–G**). Furthermore, we used ELISA to detect the serum bone transformation indicators. The results showed that DIO increased CTX-I (**Figure 2H**) and decreased PINP (**Figure 2I**) abundance. In conclusion, DIO effectively altered bone morphology, attenuated the mechanical properties of the femurs, and inhibited bone formation.

### DIO significantly changes the species abundance of gut microbiota

**Figure 3:**
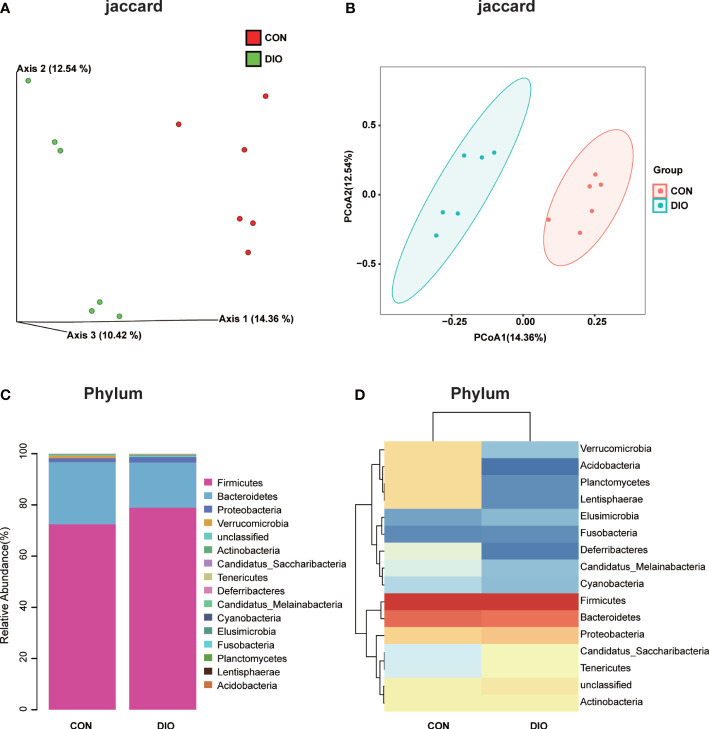
DIO significantly alters the abundance of gut microbiota. **(A, B)** 3D and 2D PCoA of GM analyzed with Jaccard distance matrices, n = 6. **(C)** Stacked bar chart at the phylum-level in the CON and DIO groups, n = 6. **(D)** Heat map at the phylum-level in the CON and DIO groups, n = 6.

A Venn diagram of the ASV distribution revealed changes in the microbiota in the DIO group. We generated 7,020 ASVs from the CON and DIO groups, including 211 differential ASVs, out of which DIO had 3,342 unique ASVs, CON had 2,753 unique ASVs, while 925 ASVs were shared between both groups (**Figure S1A**; **Table S1**). We then performed an alpha diversity analysis of the Chao1 and Shannon indices to assess the sample species richness and sequencing depth (**Figures S1B, C**). The rarefaction curve can directly reflect the rationality of sequencing data and indirectly reflect species richness in samples. When the curve tends to be flat, it shows the sequencing depth is reasonable. (**Figure S1D, E**). We then assessed the beta diversity, which reflects species differentiation between the two groups. To that end, we performed a Principal Coordinate Analysis (PCoA) to observe differences in the microbiota between the CON and DIO groups. Each point in the resulting graph represents an independent sample; the closer two points are to each other, the more similar they are. Based on the 3D and 2D results of the PCoA, analyzed based on Jaccard distance matrices, the GM in the CON and DIO groups were divided into two distinct groups, indicating that the composition of the GM in the DIO group differed significantly from that in the CON group (P<0.05) (**Figures 3A, B**).

**Figure 4:**
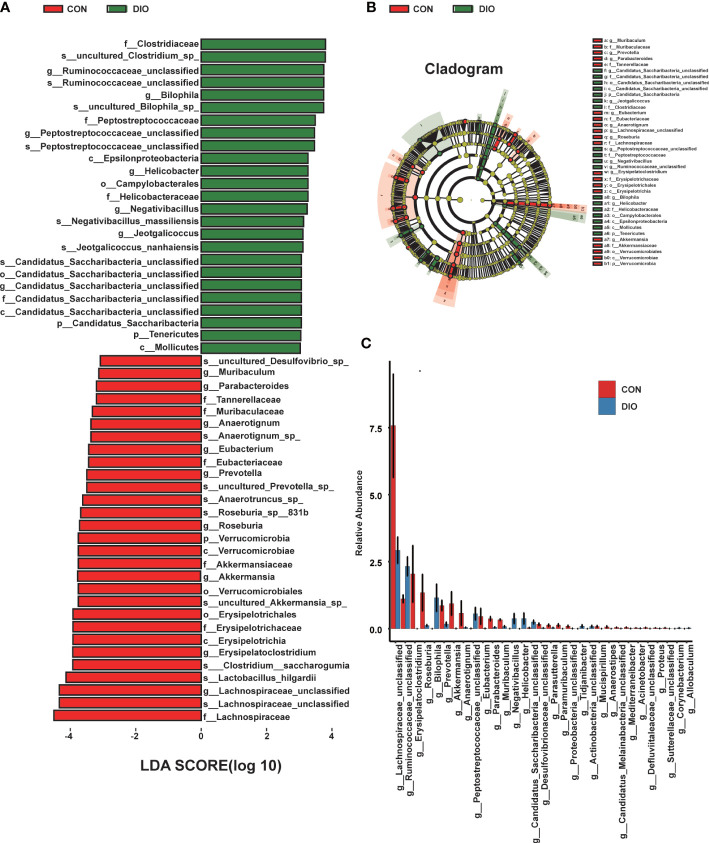
DIO changes the gut microbiota from the phylum to species levels. **(A)** LEfSe analysis of gut microbiota in the CON and DIO groups, n = 6, LDA score > 4.0. Red represents increased microbiota in the CON group; green represents increased microbiota in the DIO group. **(B)** The relationships among different microbiota from the phylum to species levels are shown in the cladogram, n = 6. **(C)** Significant difference analysis at genus level, n = 6, Wilcox test P-value < 0.05.

Next, we generated stacked bar charts and heat maps of the phylum, class, order, family, genus, and species levels to conduct a species analysis. The phylum-level analysis is shown in **Figures 3C, D**, while the results of the other levels are listed in **Figure S2**. Firmicutes and Bacteroidetes were identified as the main phyla of the GM, accounting for 80% of the total microbiome. In our study, the abundance of Firmicutes was higher in DIO, and the abundance of Bacteroidetes was lower (**Figures 3C, D**). To assess the significant differences in the abundance of the species between the two groups, we performed a linear discriminant analysis (LDA) effect size (LEfSe) analysis with an LDA fold of four. The relationships between the different microbiota from the phylum to species levels are shown in the cladogram in **Figures 4A, B**. We then performed a relative abundance analysis at each level and observed 34 species with significant differences at the genus level, out of which 13 were upregulated and 21 were downregulated (**Figure 4C**).

### DIO markedly alters the fecal metabolome

**Figure 5:**
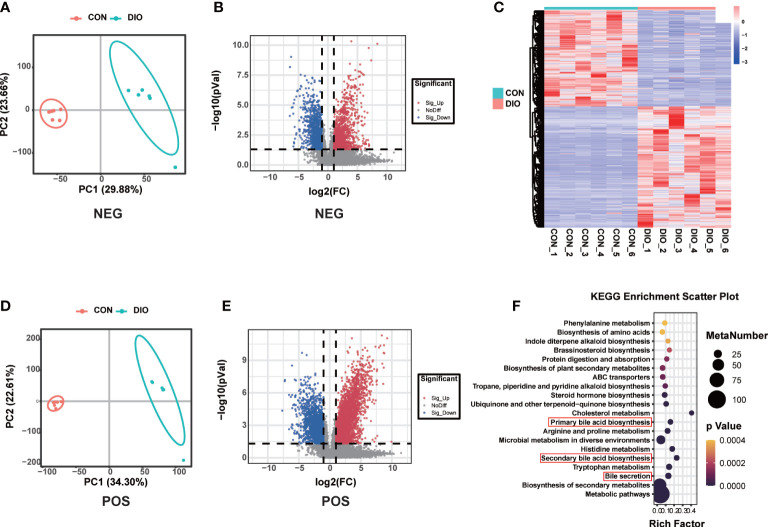
DIO markedly alters the fecal metabolome. **(A, B)** PLS-DA analysis of fecal metabolites between the two groups; variable importance in projection (VIP) > 1, R2 > 0.5, Q2 > 0.5. **(C, D)** Volcano maps displaying differential fecal metabolites. **(E)** Heat map of fecal metabolites in the two groups. **(F)** Bubble diagram of the top 20 enriched KEGG pathways.

Principal Component Analysis (PCA) was mainly used to observe the trend of separation between groups in experimental models and whether there were outliers, and to reflect the degree of variation between and within groups from the raw data. A PLS-DA was performed to identify the differences in the fecal metabolites between the two groups. Different from PCA, the PLS-DA is a supervised analysis that maximizes the differences between two groups using partial least squares regression to model the relationship between metabolite expression and sample type. It can further be used to assess sample modeling and prediction. R2 is the interpretation rate of the model in the Y direction, and Q2 is the prediction rate of the model. We tested the R2 and Q2 model parameters 200 times, the values of which are shown in **Figure S3**. The PCA and PLS-DA results revealed that the metabolite profiles of the two groups were divided into two distinct zones, indicating that DIO can alter fecal metabolites (**Figures 5A, B**, **S4**). Compared with the positive ion mode in the CON group, we identified 4,579 metabolites with alterations in the DIO group (2,620 upregulated and 1,959 downregulated), and in negative ion mode, we identified 2,110 metabolites with alterations (1,111 upregulated and 999 downregulated) (**Figures 5C, D**). As shown in the differential metabolite heat map of the comparison group, there were significant cumulative differences in the metabolites between the two groups (**Figure 5E**), among which 857 were annotated based on the KEGG database. We performed a KEGG enrichment analysis of the differential fecal metabolites between the two groups, the top 20 of which have been listed in **Figure 5F**. Among the top ten metabolic pathways, we assessed primarily those related to bile, including primary and secondary bile acid biosynthesis and bile secretion (**Figure 5F**). Compared with the CON group, 16 differential metabolites were enriched in the above three metabolic pathways in the DIO group, namely, chenodeoxyglycocholate, bilirubin, cholic acid, glycochenodeoxycholic acid, glycocholic acid, deoxycholic acid, zalcitabine, lithocholic acid (LCA), cortisol, taurochenodeoxycholic acid, taurocholate, fexofenadine, fluvastatin, taurine, 5-beta-cyprinolsulfate, and beta-muricholic acid, of which 15 were upregulated and one was downregulated. LCA was particularly interesting to identify as it is the main component of bile acids and closely related to bone metabolism (Ruiz-Gaspà et al., 2020B).

### Correlations between differential gut microbial genera and bone phenotypes

**Figure 6:**
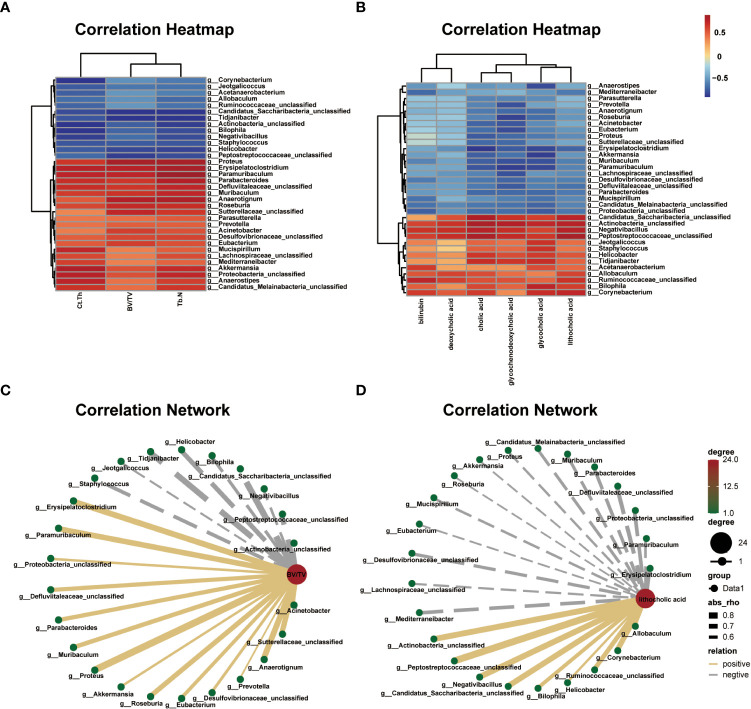
Correlations among differential gut microbial genera, bone phenotypes, and fecal metabolites related to bile acid metabolism. **(A)** Correlation heat between differential bacterial genera and bone phenotypes in the two groups, |r| > 0.5. Red represents positive correlations, and blue represents negative correlations. The darker the color, the stronger the correlation. **(B)** Correlation network map between BV/TV and differential bacterial genera, |r| > 0.5. **(C)** Correlation heat map between the differential bacterial genera and fecal metabolites related to bile acid metabolism in the two groups, |r| > 0.5. Red represents positive correlations, and blue represents negative correlations. The darker the color, the stronger the correlation. **(D)** Correlation network map between lithocholic acid (LCA) and differential bacterial genera, |r| > 0.5.

The correlation heat map and network of differential bacterial genera and bone phenotypes are plotted in **Figures 6A, B** (|r| > 0.5). Among the differential bacterial genera that were significantly related to BV/TV (|r| > 0.5), 24 of 34 distinct genus were correlated with BV/TV, including 9 negatively correlated and 15 positively correlated. The nine bacterial genera that were negatively correlated with BV/TV were *Jeotgalicoccus* (r = –0.549, P = 0.064), *Negativibacillus* (r = –0.591, P = 0.042), *Bilophila* (r = –0.623, P = 0.030), *Actinobacteria* (r = –0.630, P = 0.027), *Staphylococcus* (r = –0.657, P = 0.020), *Helicobacter* (r = –0.717, P = 0.008), *Peptostreptococcaceae* (r = –0.751, P = 0.004), *Tidjanibacter* (r = –0.795, P = 0.002) and *Candidatus Saccharibacteria* (r = –0.818, P = 0.002). The 15 bacterial genera that were positively correlated with BV/TV were *Proteus* (r = 0.865, P = 0.001), *Anaerotignum* (r = 0.797, P = 0.003), *Erysipelatoclostridium* (r = 0.794, P = 0.002), *Paramuribaculum* (r = 0.775, P = 0.003), *Sutterellaceae* (r = 0.761, P = 0.004), *Roseburia* (r = 0.741, P = 0.008), *Muribaculum* (r = 0.706, P = 0.013), *Parabacteroides* (r = 0.692, P = 0.016), *Defluviitaleaceae* (r = 0.690, P = 0.013), *Eubacterium* (r = 0.651, P = 0.021), *Acinetobacter* (r = 0.647, P = 0.023), *Desulfovibrionaceae* (r = 0.609, P = 0.035), *Proteobacteria* (r = 0.583, P = 0.046), *Akkermansia* (r = 0.576, P = 0.049), and *Prevotella* (r = 0.531, P = 0.079).

### Association analysis of differential gut microbiota and fecal metabolome

The correlation heat map and network of differential bacterial genera and fecal metabolites related to bile acid metabolism are plotted in **Figures 6C, D** (|r| > 0.5). Multiple correlations between the differential bacterial genera and fecal metabolites were identified. For example, LCA was positively correlated with *Peptostreptococcaceae* (r = 0.854, P = 0.001), *Negativibacillus* (r = 0.781, P = 0.002), *Actinobacteria* (r = 0.780, P = 0.002), *Candidatus Saccharibacteria* (r = 0.769, P = 0.005), *Corynebacterium* (r = 0.750, P = 0.004), *Ruminococcaceae* (r = 0.692, P = 0.015), *Bilophila* (r = 0.639, P = 0.025), *Allobaculum* (r = 0.624, P = 0.030), and *Helicobacter* (r = 0.545, P = 0.066), and negatively correlated with *Erysipelatoclostridium* (r = –0.705, P = 0.010), *Defluviitaleaceae* (r = –0.661, P = 0.019), *Proteobacteria* (r = –0.633, P = 0.026), *Mediterraneibacter* (r = –0.626, P = 0.029), *Parabacteroides* (r = –0.615, P = 0.037), *Desulfovibrionaceae* (r = –0.602, P = 0.038), *Muribaculum* (r = –0.594, P = 0.045), *Candidatus Melainabacteria* (r = –0.588, P = 0.044), *Paramuribaculum* (r = –0.572, P = 0.051), *Mucispirillum* (r = –0.556, P = 0.060), *Proteus* (r = –0.542, P = 0.068), *Lachnospiraceae* (r = –0.538, P = 0.074), *Akkermansia* (r = –0.512, P = 0.088), *Eubacterium* (r = –0.507, P = 0.091), and *Roseburia* (r = –0.503, P = 0.098). Further, we analyzed the correlation between bone phenotypes and differential metabolites related to bile acid and found a negative correlation between LCA and bone phenotypes (**Figure S5**).

## Discussion

GM play an indispensable role in host physiology, including nutrient absorption, immune system modulation, and homeostasis (Belkaid and Hand, 2014). GM composition is influenced by several internal and external factors, including diet, age, disease, and lifestyle (Sędzikowska and Szablewski, 2021). Imbalances in the GM, known as dysbiosis, often induce aberrant immune responses, which in turn disrupt the local and systemic homeostasis of the host leading to various disorders (Holmes et al., 2020). Increasing evidence has suggested that GM further play an important role in bone homeostasis (Ohlsson and Sjögren, 2015). The notion that the gut microbiome is a BMD regulator in health and disease is supported by an established correlation between microbiome diversity and osteoporosis (Wang et al., 2017). Extensive research has demonstrated that the effects of the GM and their metabolites in bones are secondary to immunomodulatory responses, and the perturbation of the GM can drive skeletal deterioration in pathophysiological states (Wu et al., 2010; Li et al., 2016; Li et al., 2019). However, the relationship between intestinal microorganisms and host metabolites in DIO has seldom been investigated. In this study, we analyzed the characteristics and relationships between the microbiome and fecal metabolome in DIO using a multi-omics correlation network approach.

In the 16S rDNA gene sequencing analysis, we observed that DIO was associated with GM dysbiosis. Although alpha diversity was similar between the two groups, the beta diversity was obviously different. Higher Firmicutes and lower Bacteroidetes abundance were observed in the DIO group, which is consistent with the changes reported in postmenopausal osteoporosis (Ma et al., 2020b; Wen et al., 2020) and high-fat diet-induced bone loss (Lu et al., 2021), and contrary to variations in glucocorticoid- (Li et al., 2020) and alcohol-induced osteoporosis (Cheng et al., 2021). Additionally, the abundance of *Verrucomicrobia* was decreased in DIO. Sun also observed this change in ovariectomized osteoporotic rats (Sun et al., 2021).

We further performed a correlation analysis between the bacterial genera and bone phenotypes and found that various bacterial genera were positively or negatively correlated with BV/TV (|r| > 0.5), including *Proteus*, *Erysipelatoclostridium*, *Akkermansia*, and *Roseburia*. *Proteus*, a gram-negative pathogenic bacterium, contributes to the inhibition of osteoclast formation and bone resorption by its outer-membrane vesicles (Wang et al., 2022). *Erysipelatoclostridium*, which was found to be linked to BMD at multiple sites, has been reported to produce acetate, which is a short-chain fatty acid that inhibits inflammatory Th17 cell activation and promotes Treg cell differentiation (Zhang et al., 2016; Cao et al., 2021). Th17 cells are primarily responsible for initiating and stimulating bone resorption, while Treg cells are associated with bone resorption inhibition (Srivastava et al., 2018). *Akkermansia* of the phylum *Verrucomicrobia* is a member of the GM and plays a beneficial role in the prevention of metabolic disorders (Keshavarz Azizi Raftar et al., 2021). *Akkermansia* is also beneficial in the maintenance of bone mass and strength and is likely a critical mediator of child GM-induced anti-osteoporotic effects (Liu et al., 2021). *Akkermansia* can further induce adaptive intestinal immune responses under homeostatic conditions (Ansaldo et al., 2019). Benthe found that supplementation with *Akkermansia* could decrease the presence of B cells in the colon and that mature and immature B cell frequencies in the bone marrow were increased (van der Lugt et al., 2019). B cells are the main source of osteoprotegerin in the bone microenvironment and directly participate in the regulation of bone resorption. Activated B cells overexpress the receptor activator of nuclear factor kappa B ligand (RANKL), which promotes bone resorption (D'Amelio, 2013). Another bacterium, *Roseburia*, can decrease serum levels of proinflammatory cytokines and inhibit the activation of the nucleotide-binding oligomerization segment-like receptor family 3 (NLRP3) inflammasome in murine colitis (Wu et al., 2020). The NLRP3 inflammasome, which is a new target for the prevention and control of osteoporosis, can promote bone resorption and inhibit osteogenesis (Jiang et al., 2021). Overall, DIO can lead to dysbacteriosis in the GM, and it is speculated that osteoporosis induced by different factors may represent various dysbacteriosis. GM dysbiosis may further play a key role in contributing to bone loss in DIO.

Gut-derived bacterial metabolites regulate distant organs, thereby bridging the gap between the GM and skeletal system (Zaiss et al., 2019). Thus, we performed a metabolomic analysis to explore the metabolism in DIO and clarify the pathogenic mechanisms by which GM regulate bone metabolism. Metabolomics, which has traditionally been studied with the aim of identifying biomarkers for the diagnoses and prognoses of various diseases, has been redefined from a simple biomarker identification tool to a technology for the discovery of active drivers of biological processes (Rinschen et al., 2019). Yachida performed fecal metagenomic and metabolomic studies on samples from 616 patients who underwent colonoscopies and found that colorectal cancer progression may be influenced by the metabolic output of the entire microbiota (Yachida et al., 2019). Zhang further used metabolomics profiling and found that bile acid metabolism was impaired following the administration of a high-cholesterol diet. This promoted non-alcoholic fatty liver disease-associated hepatocellular carcinoma development (Zhang et al., 2021). In our study, fecal metabolites in DIO differed significantly from those in the CON group. We also found that bile acid metabolism was abnormally active. Based on these results, we hypothesize that DIO can activate bile acid metabolism and secretion, which are involved in the regulation of bone homeostasis.

Bile acids are important for GM and bone homeostasis. They are synthesized from cholesterol in the liver and form the major components of bile, including primary and secondary bile acids (Long et al., 2017). Bile acids are significantly modified in the gut by bacterial enzymes (Joyce and Gahan, 2016). A mechanistic link between GM composition and host physiology is thought to occur *via* microbiologically-produced secondary bile acids (Pushpass et al., 2021). The functional roles of bile acids as key pleiotropic signaling mediators in metabolism and inflammation have been identified through the discovery of the G protein-coupled bile acid receptor TGR5, farnesoid X receptor, vitamin D receptor (VDR), and pregnane X receptor (Thomas et al., 2008; de Aguiar Vallim et al., 2013). The degree of bile acid receptor activation is largely influenced by the GM (Jones et al., 2014). Vitamin D plays an important role in bone metabolism and the prevention of multifactorial pathologies, including osteoporosis (Marozik et al., 2021). Vitamin D and its active metabolites further participate in bone tissue mineralization, maintaining calcium homeostasis, and bone remodeling, which are mediated through the VDR (Banjabi et al., 2020). Recent research has shown that osteoporosis and vitamin D deficiencies are common complications in patients with chronic liver disease, especially end-stage disease and chronic cholestasis (Assis, 2018; Guañabens and Parés, 2018; Ishizawa et al., 2018). LCA, which is a secondary bile acid produced by intestinal bacteria, acts as an additional physiological VDR ligand (Ishizawa et al., 2018). VDR can be activated by LCA; the degree of activation is largely influenced by the GM (Jones et al., 2014). VDR activation by LCA decreases vitamin D signaling and induces vitamin D insufficiency or deficiency by inducing vitamin D catabolism (Ishizawa et al., 2018). In addition to catabolizing vitamin D, LCA reduces the expression of two other genes, including osteocalcin, which is closely associated with bone formation, and RANKL, which is expressed in osteoblasts and regulates osteoclast formation (Li et al., 2019). The principal physiological effect of vitamin D is in the enhancement of calcium absorption in the upper intestines (Holick, 2007). Vitamin D deficiencies and insufficiencies further cause rickets and osteomalacia and are associated with increased risks of osteoporosis, cancer, autoimmune disease, infection, cardiovascular disease, obesity, and diabetes (Rosen et al., 2012). Interestingly, in the present study, LCA was significantly increased in the DIO group compared to the CON group. Previous studies have further demonstrated the deleterious consequences of LCA and bilirubin on osteoblastic cells (Ruiz-Gaspà et al., 2020a; Ruiz-Gaspà et al., 2020b). Low bone formation due to diminished osteoblast activity is the main cause of bone loss (Guañabens et al., 1990). Furthermore, we conducted a correlation analysis between metabolites and microbiota to better understand their crosstalk. We found that LCA was correlated with multiple differential bacterial genera including *Erysipelatoclostridium*, *Proteus*, *Akkermansia*, and *Roseburia*. These bacterial genera, which are significantly downregulated in DIO, are closely related to bone metabolism. Therefore, bile acid dysfunction may play a vital role in DIO pathogenesis. LCA may further be a key metabolite in DIO.

Despite our findings, our study had various limitations. Although a correlation was found between GM, fecal metabolites, and DIO, the causal and regulatory relationships among them were not clarified. Furthermore, we identified three metabolic pathways related to amino acids among the top ten pathways apart from those related to bile acid metabolism. This was noteworthy as amino acids are important energy sources in bone remodeling and affect bone resident cells through neuronal and hormonal mechanisms. These findings should therefore be further explored in future research. Our sample size was also small, and high-quality biomarkers require rigorous studies with adequate sample sizes. Therefore, a larger sample size should be used in future studies to identify potential novel biomarkers for DIO.

## Conclusion

Our study demonstrated close correlations among GM, fecal metabolites, and DIO. DIO-induced alterations in the abundance and function of GM may represent key factors affecting bone homeostasis. Our findings provide new insights into the pathogenesis of DIO and may ultimately contribute to improving diagnostic and therapeutic options for patients.

## Data availability statement

The datasets presented in this study can be found in online repositories. The names of the repository/repositories and accession number(s) can be found in the article/**Supplementary Material**.

## Ethics statement

The animal study was reviewed and approved by Shanxi Medical University. Written informed consent was obtained from the owners for the participation of their animals in this study.

## Author contributions

YF, ZL, ZT, and XQ contributed to the conception and design of the study. XQ, WW, KZ, RZ, and LY conducted experiments and maintained the animals. XQ, XL, YP, SY, WW, RZ, and LY participated in sample collection and data analysis. CX, PL, and XS gave technical support to the experiment. XQ and ZT uploaded raw data to the NCBI database. XQ drafted the manuscript. ZT, YF, and ZL revised the manuscript. All authors contributed to the article and approved the submitted version.
