# Supplementary material for: Gut microbiota and fecal metabolic signatures in rat models of disuse-induced osteoporosis
Source: Front Cell Infect Microbiol. 2022 Dec 14;12:1018897. doi: 10.3389/fcimb.2022.1018897 (PMC9798431; doi:10.3389/fcimb.2022.1018897)
Supplement: Supplementary file 1 [file DataSheet_1.docx]

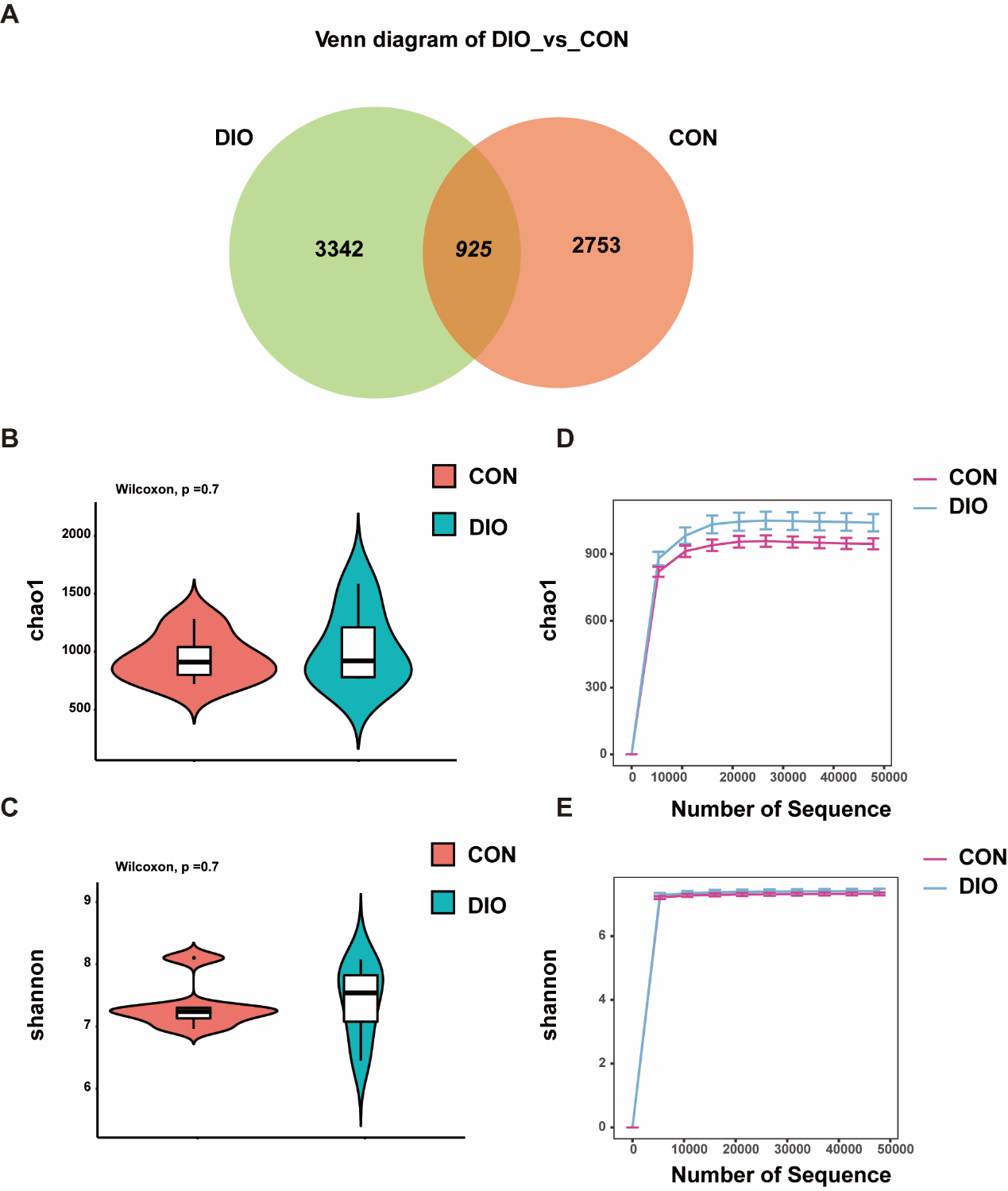


Figure S1. Alpha diversity analysis of two groups. (A) Venn diagram. (B) Chao1 index. (C) Shannon index. (D) Rarefaction curves in chao1. (E) Rarefaction curves in shannon. n = 6.

**A B**


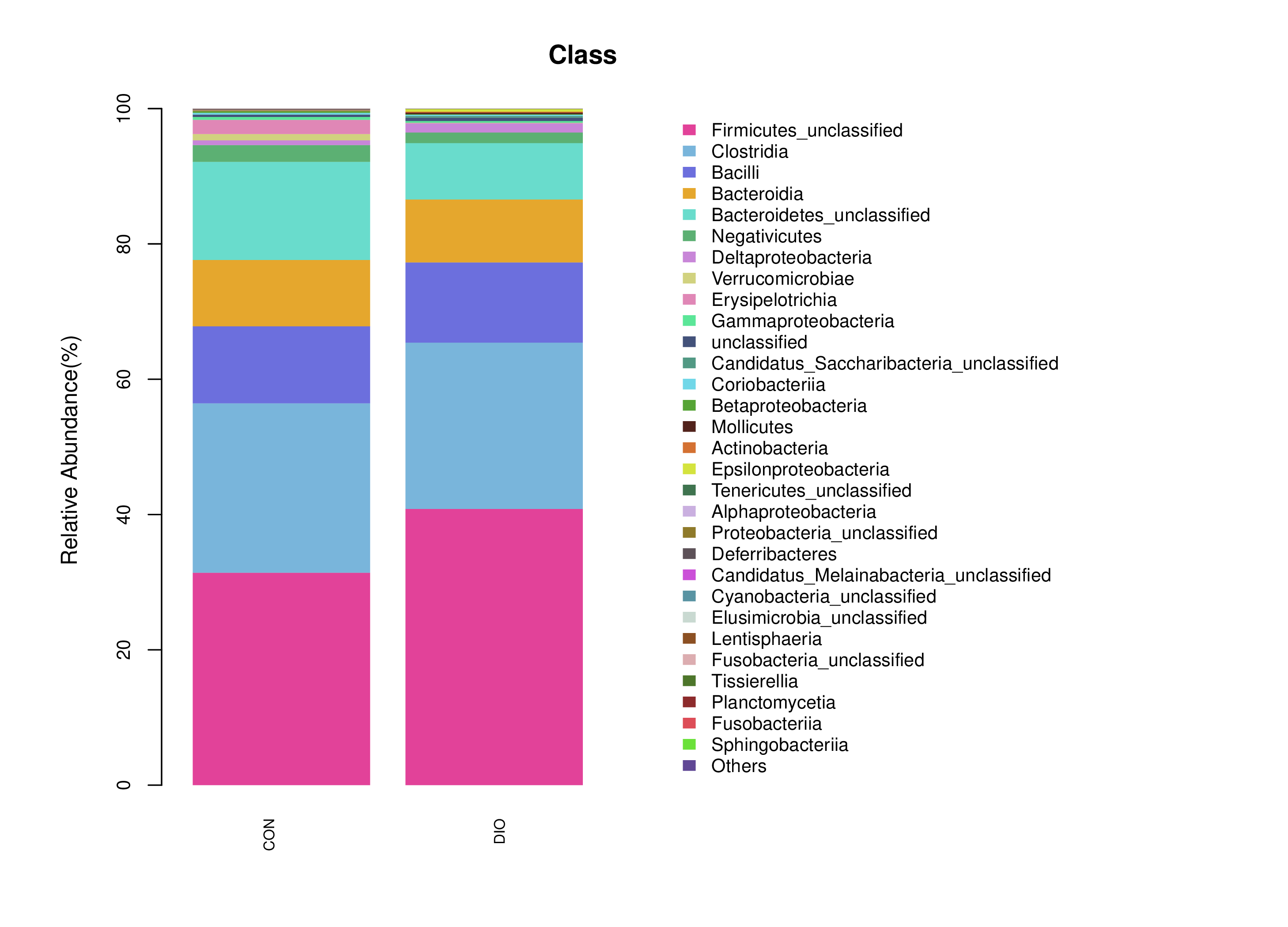

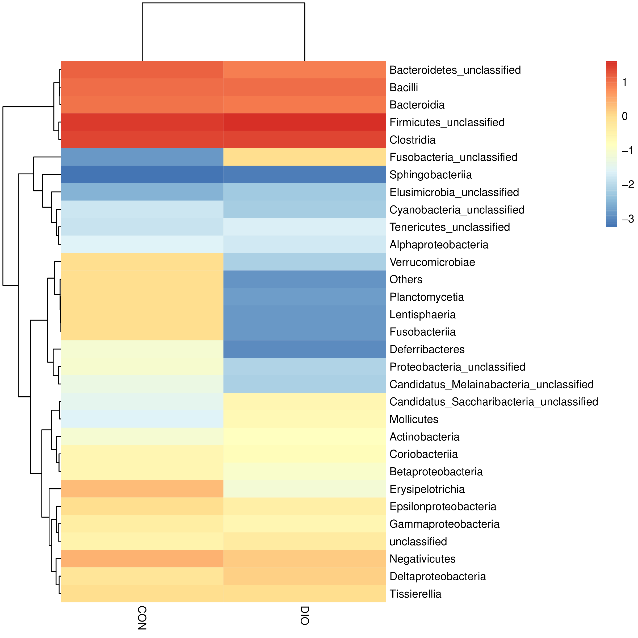


**C D**


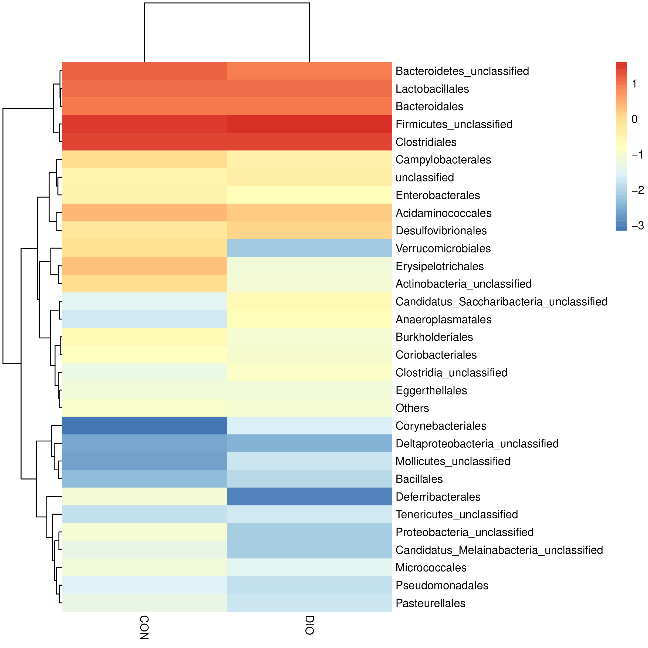

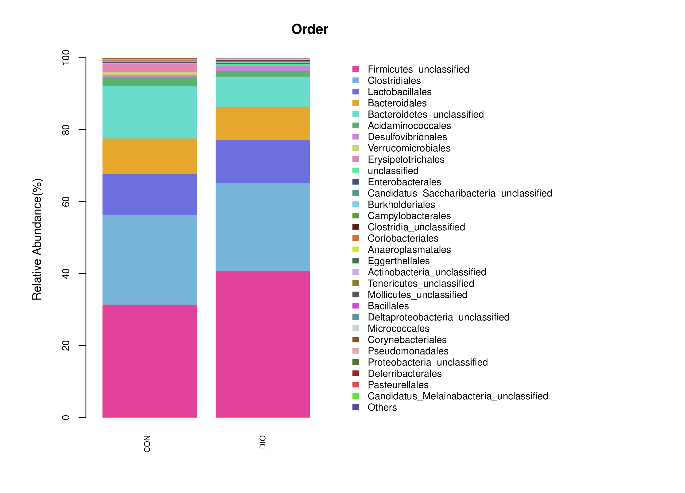


**E F**


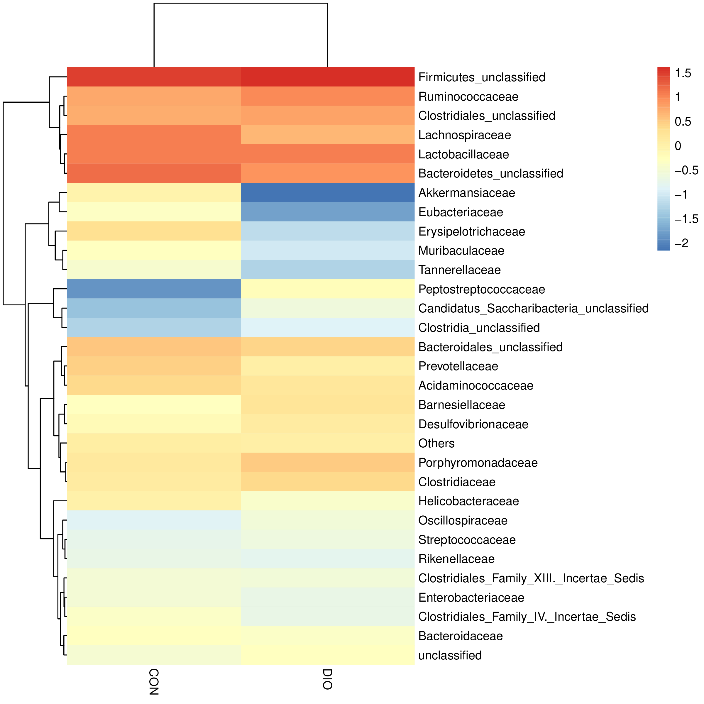

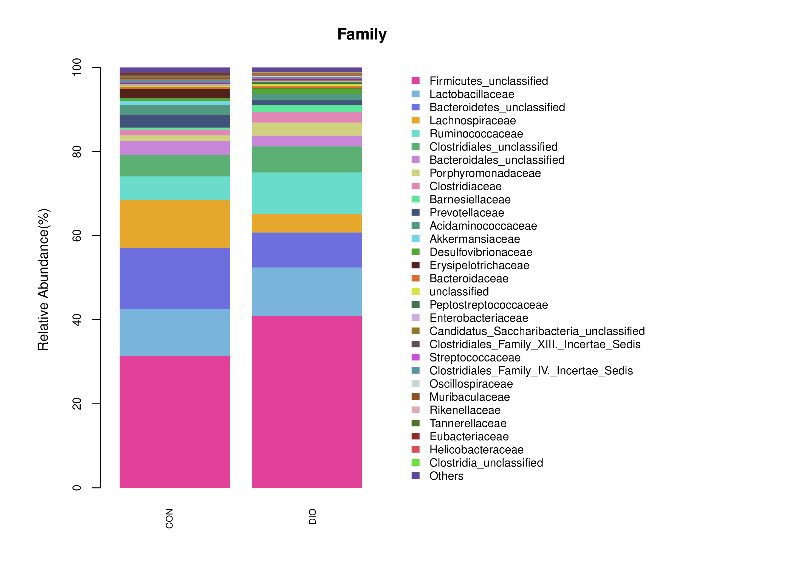


**G H**


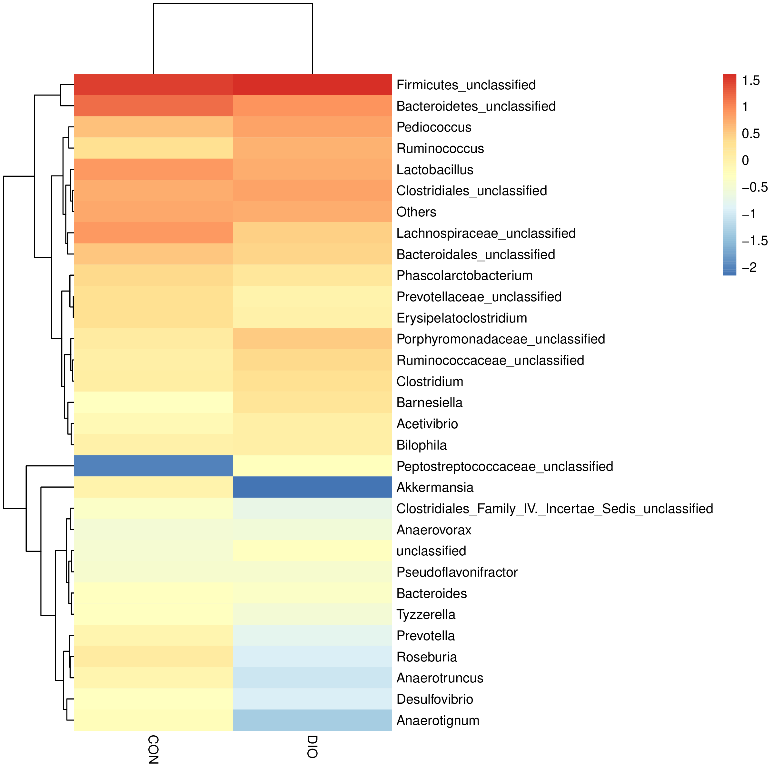

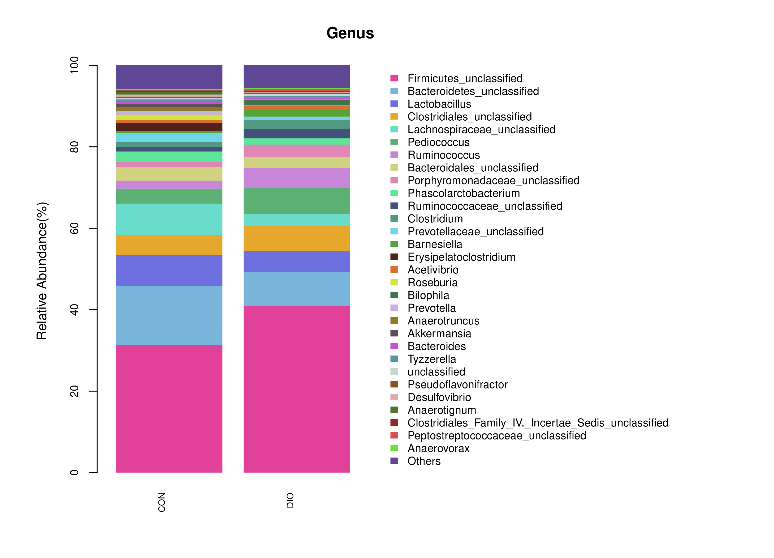


**I J**


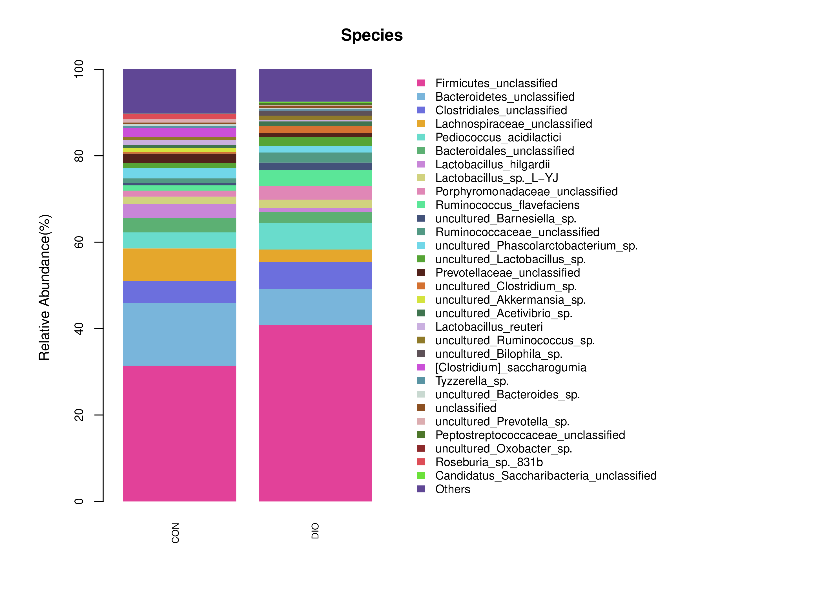


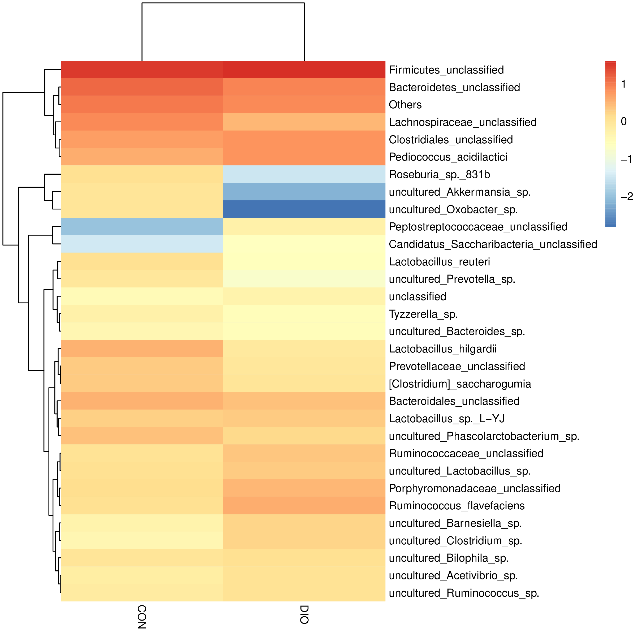


Figure S2. The stacked bar charts and heat maps of other levels between the two groups. (A, B) class-level. (C, D) order-level. (E, F) family-level. (G, H) genus-level. (I, J) species-level. n = 6.


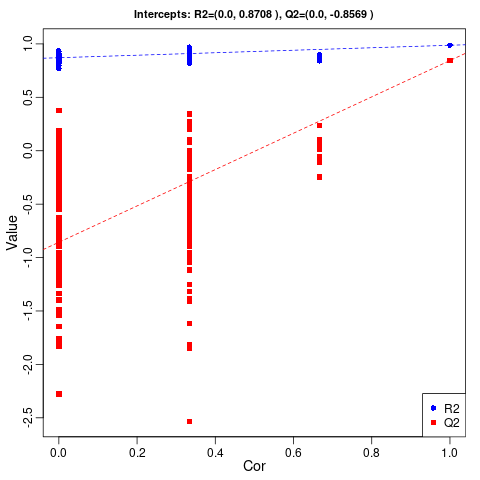
 **A**

NEG


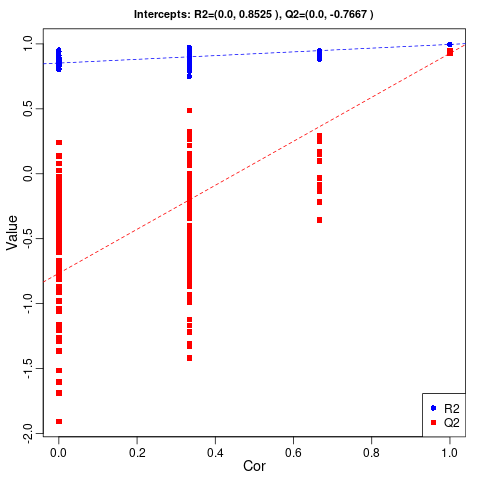
 **B**

POS

Figure S3. The R2 and Q2 model parameters in NEG and POS ion mode. (A) NEG ion mode. (B) POS ion mode. n = 6.


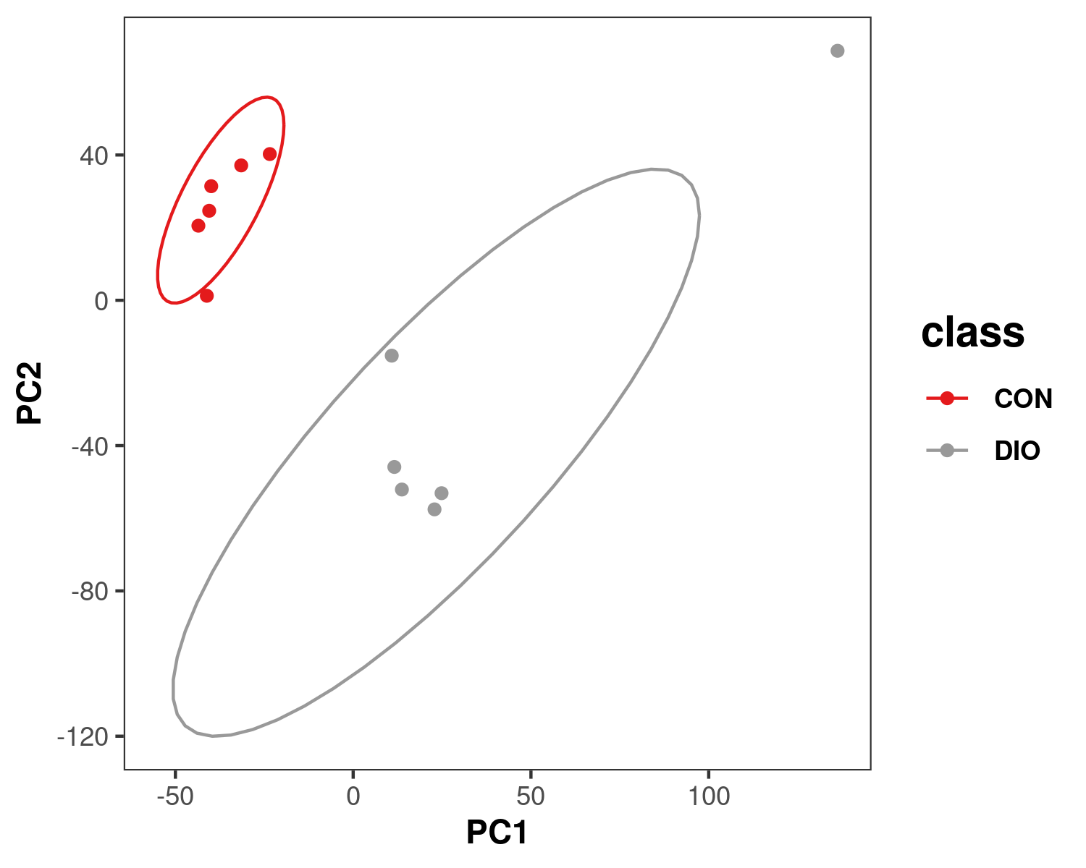
**A**

NEG


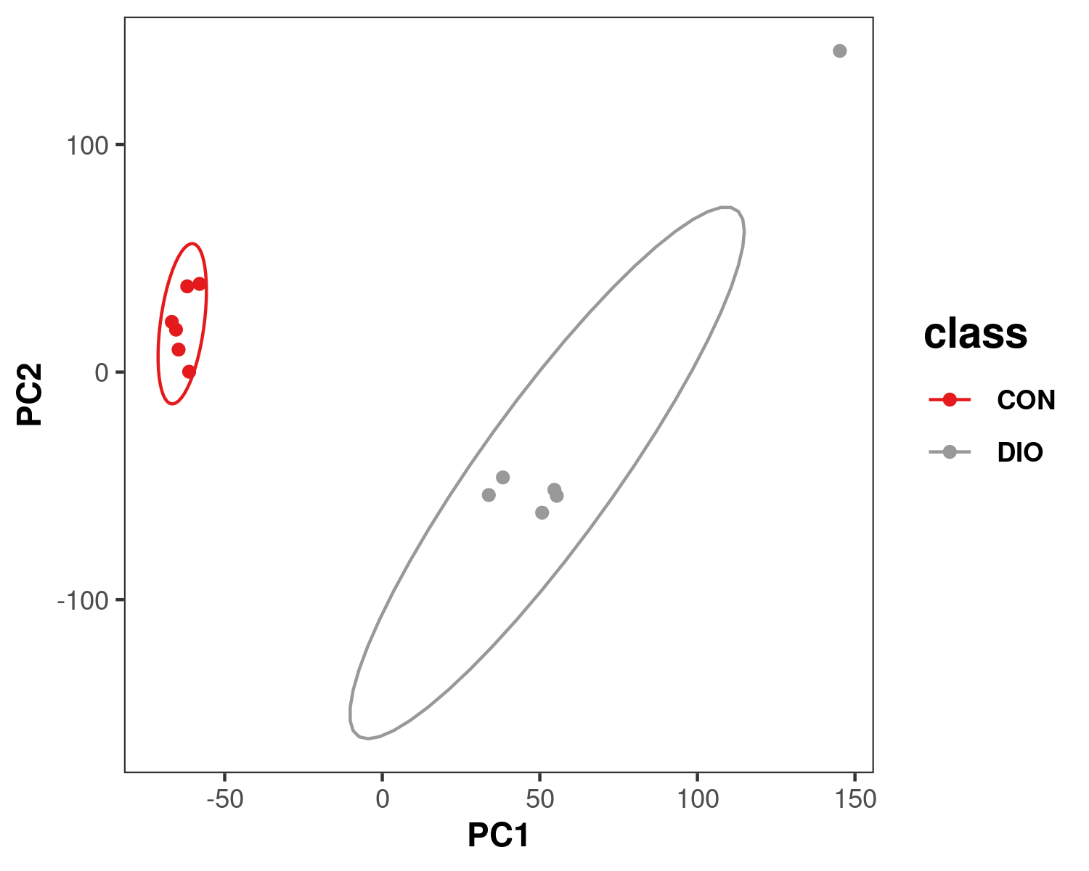
**B**

POS

Figure S4. PCA analysis in NEG and POS ion mode. (A) NEG ion mode. (B) POS ion mode. n = 6.


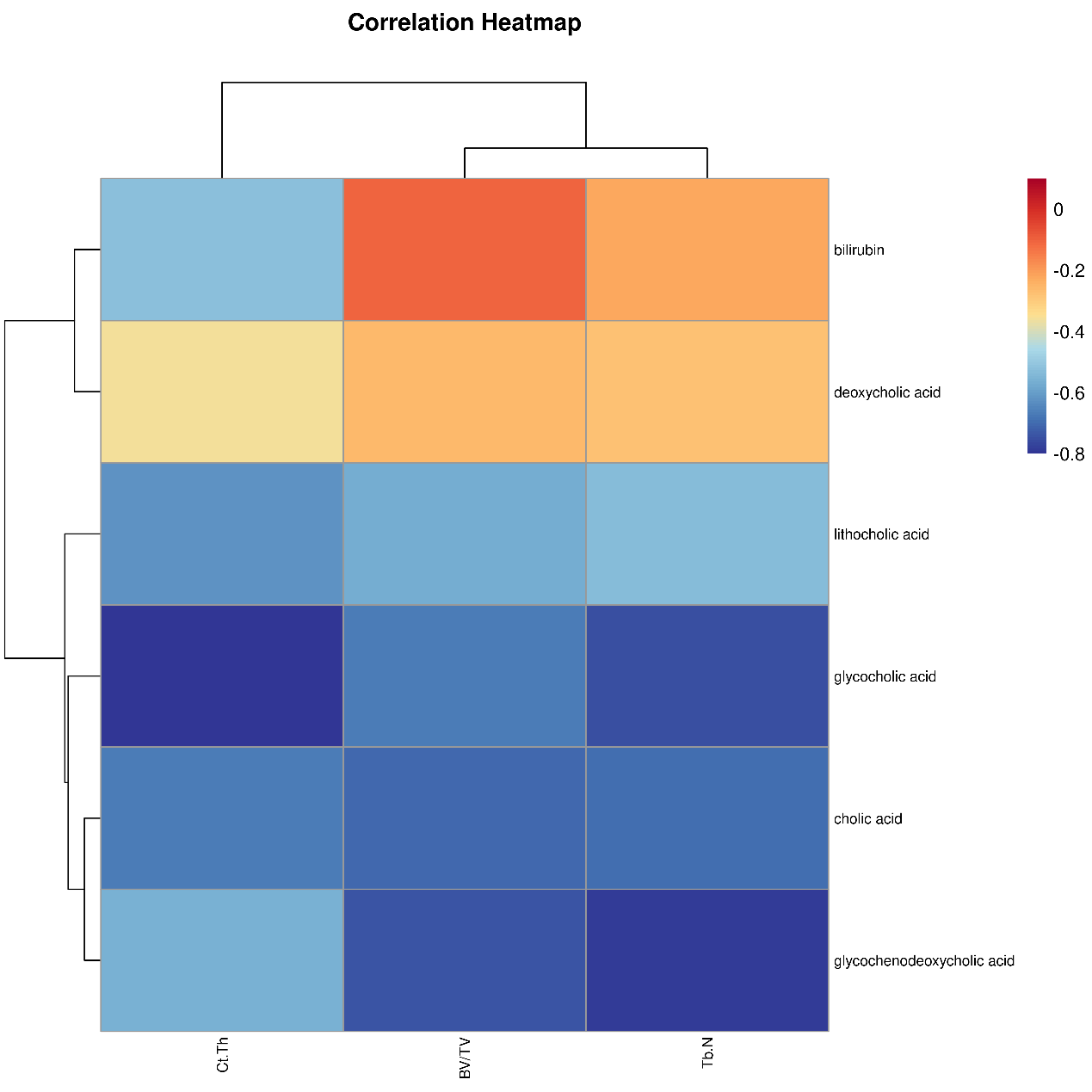


Figure S5 Correlation heat map between phenotypic data and metabolites in the two groups.

TABLE S1. ASV Level Differential Analysis

| ASV.ID | log2FC | wilcox.test.p_value | q_value | significance | regulation |
| --- | --- | --- | --- | --- | --- |
| 500b2cdb98de9094581f65ee625270c3  7d2fc27a2380015b8ea9d56b4879c6af  58032c8eed624c2156cf18daec21decf  1008871f7871f8b3cb42905ffe0807b2  d799f0364422f60ba201ee800f6a4c33  49daf16fe1e64ff6ab6b23210ed55651  58700f6d8d92310a7656ca6406f098b4  d3f43c63f8b151869e7a1d90e1679d4f  f1181f131308228a2493a9ae775a31b2  de1c0b4bb0e6cffee801a075281d5ccc  a83072dd17241cf5d580e98e03add2fa  878fd5ad92e2f7bea2881e6df1f9bdda  cf228dbae7689f3ecb1833e896e69d78  d49dbc8289cbf7ee4dda8696f9613e38  c21442e67adfd3b0f1e28af2ee14e302  e3b810c26fca6202c8475e10a03d5ea0  7794d8f9446efa6ec04a8dedd2d5196f  541aebf40b914acc51ca95bcbf5f9d07  a5eef8793ddf5fb4b141fbd69429e7c5  93485e18f31b988a3e253fd519fad692  e60a1b18a29616f8d18b108040983e13  d49c957eeaec601dc6af8a3eda911cc2  63e7a232da24973f0925541f485c809b  3ca53903eabce6ddb11f1a8b8e99c40b  99c38b92f4c6305eb99b7101f942690c  620d9972b8f99e8ca94183e2023fb7b8  6054c43b51bfaf7343eab7cc514635df  73119599aa079994481786e4e866313b  68b44dda69703d7eecfbc7d566093b15  cbb6a622a0274ec41a807fc35068c5fa  26d98efa38e835733f817b6900d10ed7  6e161811491404791fe04d0219543c5c  e6b4bddd6c72ba00d18e63e198aec7f9  47e1cd99205041c61f09de66e116df81  9cb88aea70b3a7cb0b5f4a03cf0f340b  1c602d762e9ec1da75e02bdca7e10dce  c2345c9d06a01687fd2f654340ac32b1  06bf9729a5d9c027dcb70c7b8072b6b2  ae92751ce0103cf1fe77dd7f219da00c  1a4a9237fbff0c6c9149cd9444953a8d  61067ece1f4190df929f028f27c696a5  2aa9c9e1fb21576f43e88aa0d13f7c37  1dfdcd7db65f345b41fbdf1d04386565  92f26f39aed46aa467d2d2f1c83f1934  b1201ff56e8d4c0145de260e25cc0cfd  2064f619e9d74573d10e55167ef86266  f448afad15237ade1d1461bbcd7b51be  6f1c70b6d9e037711c028629ca233185  9e20a235c547acc52759d1b7c6e32662  d8793436e349635404540abbd1a4dd09  2f4cfad9f90947b51f7d9c899583700e  b4a4548634cd696bbcf9a03847ab1456  f1ec7d603f748858a750578afebe7e2b  74cf963baa99c52fdf123fa244387846  d9c8809a3a8794709df11241e724c3f4  901a492576c8fab9d714d029f31a0047  9ed846d9c8426067f8c1e254dedbe37d  43d609757064518dbfcb7136a17e7d25  9eb6a1a0e2c613d132b4ccd395c705d0  72be040662c52a9feb4bf3a25ad0fbd2  c072170a84f5f0a62d2769579532747f  81647391b330407f43ccbe98d074df32  e37610a0684d71b812c9ed8b8e68e9d2  58df8527a14018a8ff16b6d05faecfa7  488476d16ad9bc5b1b7ed1067b1322b1  3ee466384be8a441bb986e6ccfa95778  f00ef2174545c3be94ffaea43db9a5fc  fca8463020879a0fbc9aa0e2a13cb59c  b241cf418bcc200a2cbefad8670042e2  af70d3a4d7eb9610c2c307b40c8f9db2  354f877ad63461a14cc9187df02a9998  29bd72b10db58feec8aad0dbd010a896  4adc8e3b150f8522677862845451a727  28812a25981499490dfd7fe4cc054e2a  e7f94e316126d997c98c3e6aa992c5ee  ae4748a714220ba7393f0aed19001c14  583a986f5ebf9d33038ff60eee737e06  363652c053efc170fc4c49496f117548  476c9dabf7c78d3efcd1bb90ffe414e1  f58cfb78abcdd47dff0d67d4c6e87ee0  c5c255ea3f0ea4f142ef629ebee5232f  2b2017babd79e30c7e1ded8ab4148843  dad54efeebeda9947025e0c2ab1dd31d  7922c65612d65ba2377da5bd03551725  7869db4f80989a8951685a8c13b49690  0b54e212a80a71e6b5ba436375d173d4  8a3b86465d7fca74f220e5ef28b1de34  017328e7bd29cdd29ff1e85a2cb51003  0a7909baf81962e17f5c8ab41c4f31c2  adbdd785ad200aed583f2bc39b4d4955  be3886697bb9349501f9a5a28915f43f  f470b534d6266aba45c715de60d2cad9  b7294dc27a33f510fc21538e14a93428  34073ab122a0cdcfa63561c5fd6b5a2c  7575c5d1e36ca75c5a8171a29077f1d8  75a9311d26312902306b8dbb7a802be2  b1c049aeefb630fe2937b155523c6e97  b8e1fa7c35fa1391d609df2a260fd114  e43c26563e4ea0bfaf4c3cb864d978ce  500b50c6b44d14884166bf4e56e5db08  14741c9390b18ee168e67ded65bdf097  14be37f15833760c744ea93dc597c71d  1ffd12bff44b82ca554e491b50c56195  5f737504acc11df6ccda56eee6a5abbf  197605e6cdcf48e475e6b0bda87c75ed  ccdc6fe1720d77731a424085344ede4f  b1636e9d60b5c188c6eda2303bbc4192  3023539c666867a396c8e6d2ba8b2937  16665b4b24fcea00d2ac94c908218b9a  aa8d47a2c170dae00a3e4fe02fc3e8eb  15ac158b23b7bb681ec41a0a097b02be  4dbb1858cfa1e891b27f44733c82e48d  18269d75bc8d8b04cba41f2dafbbcf28  84480a4b335049e4833b8bfa379b52b3  f16c4794342eb058f64dfd7f42cd7b62  6f3dc1313efc32cdda2ac2d36dbd97ed  833112e913f87497c57975c343a5b3d0  e4fcf96321a032bb071d7fcac03d581e  fe7328e2412c7098575d951097b9bc41  3109c1aa89878968bf9c4a1304bc25c4  9cc288377e0e08f51221f1fee309ea6a  eaa91f7a16d01406d8e3b22bd60839e6  8b167b838d7ee4117cf053898e7bd9af  768068d0790fe1e7314defebba2028dd  f287470d01dcccd57fab9160ad299338  e8ca7445f55a74aab3c89946ac1accc1  e3938d913c6c8e1eb538cfa5f5c51ff4  30aec84fa51c5735cd8e00a32742cea2  9058a12c856086b53b4b03868ab117bf  44c482d62c228f49f4a293065fd39aaa  028b7b95033fe820f2577a4d68d67801  7a1cc19d701142c07096c6917b9a627c  1ee4851d7f287a2f5e6eaf4ab2133058  441314b8d781c5dedacd1d1304f1230b  7d91203f175430765f14b752bd19c7b3  89b3bfdb50cff6aad436469f7eaaca81  2187808f8f3c2b42d8c22d0bd3ea34df  e4af4bb39f94e1a3218f3045c5548633  e7c2c6bfd9e137372d60c9bc0765c8d0  06845950cb839ada518fc1bf8717aa04  f81009c5d4f8234ccefaf52cee1b0724  f7afa0a83624a9b565aacc2580184d23  7f813743f11cbcf4157ee3883ff4c6fc  e8db76a826244b5042be629d2b409f30  3a5bc18a9562ae184c227e0d1950a006  47ef1dda9a13793b53c67270aaa8734c  d52292f6ea27f545d2484ef8b12a0e9a  aa98bca0868694ef55c26f846230829e  a53d85463ac951c4dd976bc33d125a07  ae95ae4fc59628f35757e928a7b8eba3  2a908107494c5bb728247192cdd3fa16  f2b5056dba87b6b911d80a2313a6fd0d  fe0658192b93fa6320205db953c92f86  da8c365c8f78e41350d673c40ac8e999  8c160916bba9a860adec0e3b1185330e  2b5f8194db4fc1dd35f4877291e90beb  e0ae421ffb2c71834cd0dcad1a9deda4  41f45c0ed64d083d2cc3bd5750efe9a2  6a93f2ead322c0242eec6c70c2026edd  c635f3788c00b4189314f3d20ac37f5d  fb14c079d2cb80435600c7d876e6b846  0070f5be4b0ef3f274b1daca8c546ba6  c8ba3a8296855ccb171bfca4149edc43  13dc63f66dcbe8353a597c66fa540db9  61ee4728b57e86e2588404a68fdb9fab  389fbcd230e513db0374cd11bb83221a  42c18c5d3bb88f58aef567f7d8528a00  57fa94fa640df325e8ee9337c36a834b  8a5abfdcce708b8518aa39ac2d1844d4  b07958175630f493ed7a32057e68d919  012227f79d83db6a1a52b3c1b6ba4075  78fe1b004192a9169f3a7f3be952c0ab  5524fb3f7df1fe00794b1ce2106e2a9b  dbd7328a98b2158f5eeea8507d3e556b  c20e36bbc8447046164988e25a20ef91  d22a4a07ca0d8e78f013e485bee70558  d1562e0a893b90f24a951f87ce405aea  385aeab003e1921311426e91a1668eec  bb4f9b2af18c84fcbf46bd6ebb11be78  806199bb594b4097d992d3c5572d051a  312555586fe415c688e5a9ae0314c988  c35ed2f7ac4fd424e17d64cef8108ef2  fd5d70ac0e6681a8e28aa75faaf69314  86298bbade409d463fd7c5cf006299fe  f1ef5de8ed16a5cef5e27d92111c5a91  31cdf82d26cdc7e8a54626da556926bc  61c8b3042c7346c69b5c7442428a6e2f  d6de092293052f771e2c72dac23771cd  ca7e528bcb81afc7c302e2f190f43641  405c5e55c62592fc0528fc274456efdf  0614d70ff40dce9f139ac4dfb120d454  b53457a0d3257bd9c3f27474dd074280  c6574a860c4d0c1be128deca718e53ab  b0360b32f913835b6df94a7e48872cc9  0e09c9ae0c55a7ed6d6b140a1942bc18  f2b7cba6bd4ca1a295bf71e0e75e18a9  0e8cd74fc459c9d59257c4f7bcd5dfd0  986744c91fa25a3fa736527c853122fa  bcb7c45ec879e9dcf0b8dfb73cbeef5c  1fb7175f84e41fa693be4e666f0d3298  7c9da14ba98f3a3ac3b5b3035ee91748  b47177c95f0e06ee3b38835bf596b1a1  62f69eff18887663d5bfaec55aa314c3  2f14dadc2f5e11b332546b3bffba111d  35dc6145e97da30a6f6c7675244507f6  da3460c8428aef0ec423c48c99b6f63d  3b5ecdc28df22659eef69d03a6c0a5d5  98b1a50a24af56991d0f255881438432  793bf095d075051b53c770625790b65f  7b02da976f2134d88d91a479116b4d4c  b579544bdb6aab7b4e53c3b6922d10bf | -Inf  -Inf  -Inf  -Inf  -Inf  -Inf  -Inf  Inf  -Inf  Inf  -Inf  Inf  -Inf  -Inf  -Inf  Inf  -Inf  Inf  Inf  Inf  Inf  Inf  Inf  Inf  Inf  Inf  Inf  -Inf  -8.46  -7.61  -8.25  -8.89  7.07  -6.88  8.88  -4.27  6.23  7.06  -5.86  -4.32  3.12  2.52  3.21  3.08  4.47  5.62  -2.85  2.03  -Inf  -Inf  -Inf  -Inf  -Inf  Inf  -Inf  -Inf  -Inf  -Inf  -Inf  -Inf  -Inf  Inf  -Inf  -Inf  -Inf  -Inf  -Inf  -Inf  Inf  Inf  -Inf  Inf  Inf  Inf  Inf  3.22  7.09  -5.24  -3.39  2.58  -2.69  2.33  -1.28  3.43  -3.75  6.41  7.05  -4.44  6.38  -5.06  2.88  3.87  3.67  3.30  2.75  2.64  2.10  -1.83  -1.74  4.71  -1.94  -2.24  7.74  -5.91  6.72  -2.75  2.78  3.02  -Inf  -Inf  -Inf  -Inf  -Inf  -Inf  -Inf  -Inf  Inf  -Inf  -Inf  Inf  -Inf  Inf  Inf  -Inf  -Inf  -Inf  Inf  Inf  -Inf  -Inf  -Inf  -Inf  -Inf  -Inf  -Inf  Inf  -Inf  -Inf  -Inf  Inf  Inf  -Inf  Inf  Inf  -Inf  -Inf  -Inf  -Inf  -Inf  Inf  -Inf  -Inf  -Inf  Inf  Inf  Inf  -Inf  Inf  Inf  Inf  -Inf  -Inf  -Inf  Inf  -Inf  Inf  -Inf  Inf  -Inf  Inf  Inf  -Inf  -Inf  -Inf  -Inf  -Inf  1.98  -2.81  -3.09  3.70  2  2.24  5.14  -2.40  5.63  2.62  3.65  -3.94  3.48  3.26  -1.89  1.14  -1.98  2.39  3.22  3.57  -2.42  2.21  3.62  8.77  -3.29  4.73  2.58  6.77  3.41  5.40  3.61  2.36  3.75  -3.30  4.02 | 0.00  0.00  0.00  0.00  0.00  0.00  0.00  0.00  0.00  0.00  0.00  0.00  0.00  0.00  0.00  0.00  0.00  0.00  0.00  0.00  0.00  0.00  0.00  0.00  0.00  0.00  0.00  0.00  0.00  0.00  0.00  0.00  0.00  0.00  0.00  0.00  0.00  0.00  0.00  0.00  0.00  0.00  0.00  0.00  0.00  0.01  0.01  0.01  0.01  0.01  0.01  0.01  0.01  0.01  0.01  0.01  0.01  0.01  0.01  0.01  0.01  0.01  0.01  0.01  0.01  0.01  0.01  0.01  0.01  0.01  0.01  0.01  0.01  0.01  0.01  0.01  0.01  0.01  0.01  0.01  0.01  0.01  0.01  0.01  0.01  0.01  0.01  0.01  0.01  0.01  0.01  0.01  0.01  0.01  0.01  0.02  0.02  0.02  0.02  0.02  0.02  0.02  0.02  0.02  0.02  0.02  0.02  0.02  0.02  0.02  0.02  0.02  0.02  0.02  0.02  0.02  0.02  0.02  0.02  0.02  0.02  0.02  0.02  0.02  0.02  0.02  0.02  0.02  0.02  0.02  0.02  0.02  0.02  0.02  0.02  0.02  0.02  0.02  0.02  0.02  0.02  0.02  0.02  0.02  0.02  0.02  0.02  0.02  0.02  0.02  0.02  0.02  0.02  0.02  0.02  0.02  0.02  0.02  0.02  0.02  0.02  0.02  0.02  0.02  0.02  0.02  0.02  0.02  0.02  0.02  0.02  0.02  0.02  0.02  0.02  0.02  0.02  0.02  0.02  0.02  0.03  0.03  0.03  0.03  0.03  0.03  0.03  0.03  0.03  0.03  0.04  0.04  0.05  0.05  0.05  0.05  0.05  0.05  0.05  0.05  0.05  0.05  0.05  0.05  0.05  0.05  0.05  0.05  0.05  0.05  0.05 | 0.35  0.35  0.35  0.35  0.35  0.35  0.35  0.35  0.35  0.35  0.35  0.35  0.35  0.35  0.35  0.35  0.35  0.35  0.35  0.35  0.35  0.35  0.35  0.35  0.35  0.35  0.35  0.35  0.35  0.35  0.35  0.35  0.35  0.35  0.35  0.35  0.35  0.35  0.35  0.35  0.35  0.35  0.35  0.35  0.35  0.35  0.35  0.35  0.35  0.35  0.35  0.35  0.35  0.35  0.35  0.35  0.35  0.35  0.35  0.35  0.35  0.35  0.35  0.35  0.35  0.35  0.35  0.35  0.35  0.35  0.35  0.35  0.35  0.35  0.35  0.35  0.35  0.35  0.35  0.35  0.35  0.35  0.35  0.35  0.35  0.35  0.35  0.35  0.35  0.35  0.35  0.35  0.35  0.35  0.35  0.35  0.35  0.35  0.35  0.35  0.35  0.35  0.35  0.35  0.35  0.35  0.35  0.35  0.35  0.35  0.35  0.35  0.35  0.35  0.35  0.35  0.35  0.35  0.35  0.35  0.35  0.35  0.35  0.35  0.35  0.35  0.35  0.35  0.35  0.35  0.35  0.35  0.35  0.35  0.35  0.35  0.35  0.35  0.35  0.35  0.35  0.35  0.35  0.35  0.35  0.35  0.35  0.35  0.35  0.35  0.35  0.35  0.35  0.35  0.35  0.35  0.35  0.35  0.35  0.35  0.35  0.35  0.35  0.35  0.35  0.35  0.35  0.35  0.35  0.35  0.35  0.35  0.35  0.35  0.35  0.35  0.35  0.35  0.35  0.35  0.35  0.35  0.35  0.35  0.35  0.35  0.35  0.35  0.35  0.35  0.35  0.35  0.35  0.35  0.35  0.35  0.35  0.35  0.35  0.35  0.35  0.35  0.35  0.35  0.35  0.35  0.35  0.35  0.35  0.35  0.35 | yes  yes  yes  yes  yes  yes  yes  yes  yes  yes  yes  yes  yes  yes  yes  yes  yes  yes  yes  yes  yes  yes  yes  yes  yes  yes  yes  yes  yes  yes  yes  yes  yes  yes  yes  yes  yes  yes  yes  yes  yes  yes  yes  yes  yes  yes  yes  yes  yes  yes  yes  yes  yes  yes  yes  yes  yes  yes  yes  yes  yes  yes  yes  yes  yes  yes  yes  yes  yes  yes  yes  yes  yes  yes  yes  yes  yes  yes  yes  yes  yes  yes  yes  yes  yes  yes  yes  yes  yes  yes  yes  yes  yes  yes  yes  yes  yes  yes  yes  yes  yes  yes  yes  yes  yes  yes  yes  yes  yes  yes  yes  yes  yes  yes  yes  yes  yes  yes  yes  yes  yes  yes  yes  yes  yes  yes  yes  yes  yes  yes  yes  yes  yes  yes  yes  yes  yes  yes  yes  yes  yes  yes  yes  yes  yes  yes  yes  yes  yes  yes  yes  yes  yes  yes  yes  yes  yes  yes  yes  yes  yes  yes  yes  yes  yes  yes  yes  yes  yes  yes  yes  yes  yes  yes  yes  yes  yes  yes  yes  yes  yes  yes  yes  yes  yes  yes  yes  yes  yes  yes  yes  yes  yes  yes  yes  yes  yes  yes  yes  yes  yes  yes  yes  yes  yes  yes  yes  yes  yes  yes  yes | down  down  down  down  down  down  down  up  down  up  down  up  down  down  down  up  down  up  up  up  up  up  up  up  up  up  up  down  down  down  down  down  up  down  up  down  up  up  down  down  up  up  up  up  up  up  down  up  down  down  down  down  down  up  down  down  down  down  down  down  down  up  down  down  down  down  down  down  up  up  down  up  up  up  up  up  up  down  down  up  down  up  down  up  down  up  up  down  up  down  up  up  up  up  up  up  up  down  down  up  down  down  up  down  up  down  up  up  down  down  down  down  down  down  down  down  up  down  down  up  down  up  up  down  down  down  up  up  down  down  down  down  down  down  down  up  down  down  down  up  up  down  up  up  down  down  down  down  down  up  down  down  down  up  up  up  down  up  up  up  down  down  down  up  down  up  down  up  down  up  up  down  down  down  down  down  up  down  down  up  up  up  up  down  up  up  up  down  up  up  down  up  down  up  up  up  down  up  up  up  down  up  up  up  up  up  up  up  up  down  up |

TABLE S2. Clean Data.

| Sample | Raw_Tags | Raw_Bases | Valid_Tags | Valid_Bases | Valid% | Q20% | Q30% | GC% |
| --- | --- | --- | --- | --- | --- | --- | --- | --- |
| CON1 | 83807 | 41.90M | 73350 | 30.12M | 87.52 | 97.90 | 93.78 | 53.67 |
| CON2 | 64029 | 32.01M | 59671 | 24.60M | 93.19 | 97.61 | 93.09 | 53.46 |
| CON3 | 82121 | 41.06M | 62900 | 26.02M | 76.59 | 97.90 | 93.70 | 52.79 |
| CON4 | 81106 | 40.55M | 70806 | 29.27M | 87.30 | 97.29 | 92.19 | 53.25 |
| CON5 | 81388 | 40.69M | 66281 | 27.35M | 81.44 | 97.41 | 92.45 | 53.18 |
| CON6 | 85679 | 42.84M | 72661 | 29.68M | 84.81 | 97.42 | 92.53 | 53.91 |
| DIO1 | 87855 | 43.93M | 69458 | 28.73M | 79.06 | 97.34 | 92.53 | 53.80 |
| DIO2 | 83716 | 41.86M | 75191 | 31.12M | 89.82 | 97.44 | 92.68 | 53.16 |
| DIO3 | 85629 | 42.81M | 67368 | 27.87M | 78.67 | 96.96 | 91.19 | 52.27 |
| DIO4 | 85772 | 42.89M | 70135 | 28.57M | 81.77 | 97.58 | 92.98 | 53.68 |
| DIO5 | 83651 | 41.83M | 70670 | 28.95M | 84.48 | 97.41 | 92.54 | 52.86 |
| DIO6 | 86762 | 43.38M | 79596 | 32.53M | 91.74 | 97.32 | 92.05 | 53.71 |

TABLE S3. Details of TOP20 KEGG Pathways Enrichment Analysis

| KEGG | Pathway | NumberFeature | Pvalue |
| --- | --- | --- | --- |
| map01100  map01110  map04976  map00380  map00121  map00340  map01120  map00330  map00120  map04979  map00130  map00140  map00960  map02010  map01060  map04974  map00905  map00403  map01230  map00360 | Metabolic pathways  Biosynthesis of secondary metabolites  Bile secretion  Tryptophan metabolism  Secondary bile acid biosynthesis  Histidine metabolism  Microbial metabolism in diverse environments  Arginine and proline metabolism  Primary bile acid biosynthesis  Cholesterol metabolism  Ubiquinone and other terpenoid-quinone biosynthesis  Steroid hormone biosynthesis  Tropane, piperidine and pyridine alkaloid biosynthesis  ABC transporters  Biosynthesis of plant secondary metabolites  Protein digestion and absorption  Brassinosteroid biosynthesis  Indole diterpene alkaloid biosynthesis  Biosynthesis of amino acids  Phenylalanine metabolism | 165  108  23  12  17  15  37  11  12  8  21  20  11  16  11  7  8  6  9  6 | 1.76E-35  1.16E-15  4.24E-10  1.09E-09  3.04E-09  2.86E-08  5.94E-08  1.27E-07  5.61E-07  2.36E-06  9.59E-06  9.76E-06  7.95E-05  0.000101501  0.000124046  0.000130615  0.000228736  0.000381359  0.000412079  0.000416465 |

TABLE S4. Details of metabolites in bile related pathways.

| KEGG | Pathway | Feature |
| --- | --- | --- |
| map01110  map04976  map00121 | Biosynthesis of secondary metabolites  Bile secretion  Secondary bile acid biosynthesis | chenodeoxyglycocholate, bilirubin, lithocholic acid, cholic acid, glycochenodeoxycholic acid, glycocholic acid, deoxycholic acid, zalcitabine, cortisol, taurochenodeoxycholic acid, taurocholate, fexofenadine, Fluvastatin  glycochenodeoxycholic acid, glycocholic acid, taurochenodeoxycholic acid, taurocholate, chenodeoxyglycocholate, taurine, 5beta-cyprinolsulfate, cholic acid  glycochenodeoxycholic acid, glycocholic acid, taurochenodeoxycholic acid, lithocholic acid, taurocholate, deoxycholic acid, chenodeoxyglycocholate, cholic acid, beta-Muricholic acid |

TABLE S5. Details of the LEFSE result.

| Species | log10 | group | LDA | P value |
| --- | --- | --- | --- | --- |
| d__Bacteria.p__Firmicutes.c__Erysipelotrichia.o__Erysipelotrichales.f__Erysipelotrichaceae.g__Erysipelatoclostridium.s___Clostridium__saccharogumia  d__Bacteria.p__Proteobacteria.c__Epsilonproteobacteria.o__Campylobacterales.f__Helicobacteraceae.g__Helicobacter  d__Bacteria.p__Verrucomicrobia.c__Verrucomicrobiae.o__Verrucomicrobiales.f__Akkermansiaceae.g__Akkermansia  d__Bacteria.p__Candidatus_Saccharibacteria  d__Bacteria.p__Firmicutes.c__Clostridia.o__Clostridiales.f__Lachnospiraceae.g__Roseburia.s__Roseburia_sp__831b  d__Bacteria.p__Bacteroidetes.c__Bacteroidia.o__Bacteroidales.f__Muribaculaceae.g__Muribaculum  d__Bacteria.p__Verrucomicrobia  d__Bacteria.p__Candidatus_Saccharibacteria.c__Candidatus_Saccharibacteria_unclassified  d__Bacteria.p__Firmicutes.c__Clostridia.o__Clostridiales.f__Eubacteriaceae.g__Eubacterium  d__Bacteria.p__Firmicutes.c__Clostridia.o__Clostridiales.f__Ruminococcaceae.g__Negativibacillus  d__Bacteria.p__Verrucomicrobia.c__Verrucomicrobiae.o__Verrucomicrobiales.f__Akkermansiaceae  d__Bacteria.p__Bacteroidetes.c__Bacteroidia.o__Bacteroidales.f__Prevotellaceae.g__Prevotella.s__uncultured_Prevotella_sp_  d__Bacteria.p__Bacteroidetes.c__Bacteroidia.o__Bacteroidales.f__Tannerellaceae  d__Bacteria.p__Proteobacteria.c__Epsilonproteobacteria.o__Campylobacterales.f__Helicobacteraceae  d__Bacteria.p__Proteobacteria.c__Epsilonproteobacteria.o__Campylobacterales  d__Bacteria.p__Proteobacteria.c__Deltaproteobacteria.o__Desulfovibrionales.f__Desulfovibrionaceae.g__Bilophila  d__Bacteria.p__Proteobacteria.c__Deltaproteobacteria.o__Desulfovibrionales.f__Desulfovibrionaceae.g__Bilophila.s__uncultured_Bilophila_sp_  d__Bacteria.p__Tenericutes.c__Mollicutes  d__Bacteria.p__Firmicutes.c__Bacilli.o__Bacillales.f__Staphylococcaceae.g__Jeotgalicoccus.s__Jeotgalicoccus_nanhaiensis  d__Bacteria.p__Tenericutes  d__Bacteria.p__Firmicutes.c__Erysipelotrichia.o__Erysipelotrichales.f__Erysipelotrichaceae.g__Erysipelatoclostridium  d__Bacteria.p__Proteobacteria.c__Deltaproteobacteria.o__Desulfovibrionales.f__Desulfovibrionaceae.g__Desulfovibrio.s__uncultured_Desulfovibrio_sp_  d__Bacteria.p__Firmicutes.c__Erysipelotrichia.o__Erysipelotrichales.f__Erysipelotrichaceae  d__Bacteria.p__Verrucomicrobia.c__Verrucomicrobiae.o__Verrucomicrobiales  d__Bacteria.p__Firmicutes.c__Clostridia.o__Clostridiales.f__Lachnospiraceae.g__Lachnospiraceae_unclassified.s__Lachnospiraceae_unclassified  d__Bacteria.p__Verrucomicrobia.c__Verrucomicrobiae.o__Verrucomicrobiales.f__Akkermansiaceae.g__Akkermansia.s__uncultured_Akkermansia_sp_  d__Bacteria.p__Bacteroidetes.c__Bacteroidia.o__Bacteroidales.f__Muribaculaceae  d__Bacteria.p__Candidatus_Saccharibacteria.c__Candidatus_Saccharibacteria_unclassified.o__Candidatus_Saccharibacteria_unclassified.f__Candidatus_Saccharibacteria_unclassified  d__Bacteria.p__Firmicutes.c__Clostridia.o__Clostridiales.f__Clostridiaceae.g__Clostridium.s__uncultured_Clostridium_sp_  d__Bacteria.p__Firmicutes.c__Clostridia.o__Clostridiales.f__Lachnospiraceae.g__Lachnospiraceae_unclassified  d__Bacteria.p__Candidatus_Saccharibacteria.c__Candidatus_Saccharibacteria_unclassified.o__Candidatus_Saccharibacteria_unclassified.f__Candidatus_Saccharibacteria_unclassified.g__Candidatus_Saccharibacteria_unclassified  d__Bacteria.p__Firmicutes.c__Erysipelotrichia  d__Bacteria.p__Firmicutes.c__Clostridia.o__Clostridiales.f__Ruminococcaceae.g__Anaerotruncus.s__Anaerotruncus_sp_  d__Bacteria.p__Firmicutes.c__Clostridia.o__Clostridiales.f__Lachnospiraceae  d__Bacteria.p__Proteobacteria.c__Epsilonproteobacteria  d__Bacteria.p__Firmicutes.c__Clostridia.o__Clostridiales.f__Lachnospiraceae.g__Anaerotignum.s__Anaerotignum_sp_  d__Bacteria.p__Candidatus_Saccharibacteria.c__Candidatus_Saccharibacteria_unclassified.o__Candidatus_Saccharibacteria_unclassified  d__Bacteria.p__Firmicutes.c__Clostridia.o__Clostridiales.f__Lachnospiraceae.g__Roseburia  d__Bacteria.p__Firmicutes.c__Clostridia.o__Clostridiales.f__Clostridiaceae  d__Bacteria.p__Firmicutes.c__Erysipelotrichia.o__Erysipelotrichales  d__Bacteria.p__Firmicutes.c__Clostridia.o__Clostridiales.f__Peptostreptococcaceae.g__Peptostreptococcaceae_unclassified  d__Bacteria.p__Verrucomicrobia.c__Verrucomicrobiae  d__Bacteria.p__Firmicutes.c__Clostridia.o__Clostridiales.f__Ruminococcaceae.g__Negativibacillus.s__Negativibacillus_massiliensis  d__Bacteria.p__Firmicutes.c__Clostridia.o__Clostridiales.f__Peptostreptococcaceae.g__Peptostreptococcaceae_unclassified.s__Peptostreptococcaceae_unclassified  d__Bacteria.p__Firmicutes.c__Bacilli.o__Lactobacillales.f__Lactobacillaceae.g__Lactobacillus.s__Lactobacillus_hilgardii  d__Bacteria.p__Firmicutes.c__Clostridia.o__Clostridiales.f__Peptostreptococcaceae  d__Bacteria.p__Firmicutes.c__Clostridia.o__Clostridiales.f__Ruminococcaceae.g__Ruminococcaceae_unclassified.s__Ruminococcaceae_unclassified  d__Bacteria.p__Firmicutes.c__Clostridia.o__Clostridiales.f__Lachnospiraceae.g__Anaerotignum  d__Bacteria.p__Bacteroidetes.c__Bacteroidia.o__Bacteroidales.f__Prevotellaceae.g__Prevotella  d__Bacteria.p__Bacteroidetes.c__Bacteroidia.o__Bacteroidales.f__Tannerellaceae.g__Parabacteroides  d__Bacteria.p__Firmicutes.c__Clostridia.o__Clostridiales.f__Eubacteriaceae  d__Bacteria.p__Candidatus_Saccharibacteria.c__Candidatus_Saccharibacteria_unclassified.o__Candidatus_Saccharibacteria_unclassified.f__Candidatus_Saccharibacteria_unclassified.g__Candidatus_Saccharibacteria_unclassified.s__Candidatus_Saccharibacteria_unclassified  d__Bacteria.p__Firmicutes.c__Bacilli.o__Bacillales.f__Staphylococcaceae.g__Jeotgalicoccus  d__Bacteria.p__Firmicutes.c__Clostridia.o__Clostridiales.f__Ruminococcaceae.g__Ruminococcaceae_unclassified | 4.310  3.576  3.972  3.393  4.067  3.521  3.972  3.393  3.662  3.574  3.972  3.936  3.570  3.576  3.576  4.061  4.061  3.343  1.447  3.382  4.310  3.267  4.323  3.972  4.879  3.972  3.705  3.393  4.203  4.879  3.393  4.323  3.904  5.058  3.576  3.763  3.393  4.128  4.400  4.323  3.747  3.972  3.468  3.747  4.510  3.768  4.366  3.763  3.936  3.570  3.673  3.393  1.447  4.366 | CON  DIO  CON  DIO  CON  CON  CON  DIO  CON  DIO  CON  CON  CON  DIO  DIO  DIO  DIO  DIO  DIO  DIO  CON  CON  CON  CON  CON  CON  CON  DIO  DIO  CON  DIO  CON  CON  CON  DIO  CON  DIO  CON  DIO  CON  DIO  CON  DIO  DIO  CON  DIO  DIO  CON  CON  CON  CON  DIO  DIO  DIO | 3.923  3.264  3.756  3.063  3.680  3.127  3.755  3.063  3.438  3.240  3.755  3.496  3.199  3.264  3.264  3.741  3.741  3.027  3.110  3.043  3.923  3.080  3.918  3.756  4.334  3.756  3.323  3.063  3.793  4.334  3.063  3.919  3.620  4.500  3.264  3.367  3.063  3.716  3.801  3.918  3.463  3.755  3.130  3.463  4.132  3.484  3.747  3.367  3.496  3.199  3.441  3.063  3.110  3.747 | 0.002092  0.007397  0.009105  0.006485  0.009875  0.003947  0.009875  0.006485  0.016122  0.003699  0.009105  0.016309  0.003947  0.007397  0.007397  0.007397  0.007397  0.037040  0.022229  0.037372  0.002092  0.022486  0.003947  0.009105  0.016309  0.009105  0.003947  0.006485  0.006485  0.016309  0.006485  0.003947  0.006392  0.016309  0.007397  0.016309  0.006485  0.010405  0.037372  0.003947  0.003345  0.009105  0.002092  0.003345  0.024974  0.003345  0.010405  0.016309  0.016309  0.003947  0.003947  0.006485  0.022229  0.010405 |
